# Estimation of Machine Learning–Based Models to Predict Dementia Risk in Patients With Atherosclerotic Cardiovascular Diseases: UK Biobank Study

**DOI:** 10.2196/64148

**Published:** 2025-02-26

**Authors:** Zhengsheng Gu, Shuang Liu, Huijuan Ma, Yifan Long, Xuehao Jiao, Xin Gao, Bingying Du, Xiaoying Bi, Xingjie Shi

**Affiliations:** 1 Department of Neurology First Affiliated Hospital of Naval Medical University Shanghai China; 2 KLATASDS-MOE Academy of Statistics and Interdisciplinary Sciences, School of Statistics East China Normal University Shanghai China; 3 Institute for Translational Brain Research Fudan University Shanghai China

**Keywords:** atherosclerotic cardiovascular disease, dementia, Alzheimer disease, vascular dementia, machine learning, UK Biobank

## Abstract

**Background:**

The atherosclerotic cardiovascular disease (ASCVD) is associated with dementia. However, the risk factors of dementia in patients with ASCVD remain unclear, necessitating the development of accurate prediction models.

**Objective:**

The aim of the study is to develop a machine learning model for use in patients with ASCVD to predict dementia risk using available clinical and sociodemographic data.

**Methods:**

This prognostic study included patients with ASCVD between 2006 and 2010, with registration of follow-up data ending on April 2023 based on the UK Biobank. We implemented a data-driven strategy, identifying predictors from 316 variables and developing a machine learning model to predict the risk of incident dementia, Alzheimer disease, and vascular dementia within 5, 10, and longer-term follow-up in patients with ASCVD.

**Results:**

A total of 29,561 patients with ASCVD were included, and 1334 (4.51%) developed dementia during a median follow-up time of 10.3 (IQR 7.6-12.4) years. The best prediction model (UK Biobank ASCVD risk prediction model) was light gradient boosting machine, comprising 10 predictors including age, time to complete pairs matching tasks, mean time to correctly identify matches, mean sphered cell volume, glucose levels, forced expiratory volume in 1 second *z* score, C-reactive protein, forced vital capacity, time engaging in activities, and age first had sexual intercourse. This model achieved the following performance metrics for all incident dementia: area under the receiver operating characteristic curve: mean 0.866 (SD 0.027), accuracy: mean 0.883 (SD 0.010), sensitivity: mean 0.637 (SD 0.084), specificity: mean 0.914 (SD 0.012), precision: mean 0.479 (SD 0.031), and *F*_1_-score: mean 0.546 (SD 0.043). Meanwhile, this model was well-calibrated (Kolmogorov-Smirnov test showed goodness-of-fit *P* value>.99) and maintained robust performance across different temporal cohorts. Besides, the model had a beneficial potential in clinical practice with a decision curve analysis.

**Conclusions:**

The findings of this study suggest that predictive modeling could inform patients and clinicians about ASCVD at risk for dementia.

## Introduction

Cardiovascular disease (CVD) is the leading cause of noncommunicable disease and mortality worldwide [1]. Meanwhile, the epidemiology of the atherosclerotic cardiovascular disease (ASCVD), which encompasses coronary heart disease and cerebrovascular disease (CeVD), has experienced substantial and rapid growth [2]. It is reported that in 2016, ASCVD was responsible for approximately 2.4 million deaths, representing 25% of all deaths and 61% of CVD-related deaths in China [3].

Dementia is another devastating disease affecting more than 50 million individuals worldwide [4]. Given the high costs and heavy burdens it imposes on families and society, scientists and scholars around the world are dedicated to identifying preventable interventions and reducing the incidence of dementia. Recently, a growing body of evidence indicates that lifestyle interventions early in life with a focus on reducing cardiovascular risk factors are a promising strategy for preventing dementia [5-9]. In particular, shared risk factors between dementia and ASCVD have been identified [10]. According to the Lancet Commission, it is estimated that approximately 40% of dementia cases can be prevented by targeting modifiable, primarily cardiovascular risk factors [4]. However, these studies were restricted by their use of classical statistical analyses (such as Cox or logistic regressions) and by considering only widely studied prespecified CVD risks. Therefore, the results were not sufficient in accuracy.

Machine learning (ML) is an emerging technical foundation of artificial intelligence, which enables the leverage of information from large and complex datasets [11]. Several studies have applied ML-based models to dementia diagnosis and risk prediction [12-15]. Nevertheless, the long-term risk of dementia progression (5 or 10 years) in patients with ASCVD remains uncertain.

In this study, we used comprehensive phenotypic and follow-up data from a cohort of over 500,000 UK Biobank participants to develop an ML-based model capable of predicting the 5- or 10-year risk of incident dementia in specific patients with ASCVD. We anticipate that this ML-derived early warning system will enhance clinician-patient counseling, enable targeted follow-up, and facilitate the development of personalized prevention strategies. This, ultimately, can optimize the health and care of individuals with ASCVD.

## Methods

### Data Source and Study Population

**Figure 1:**
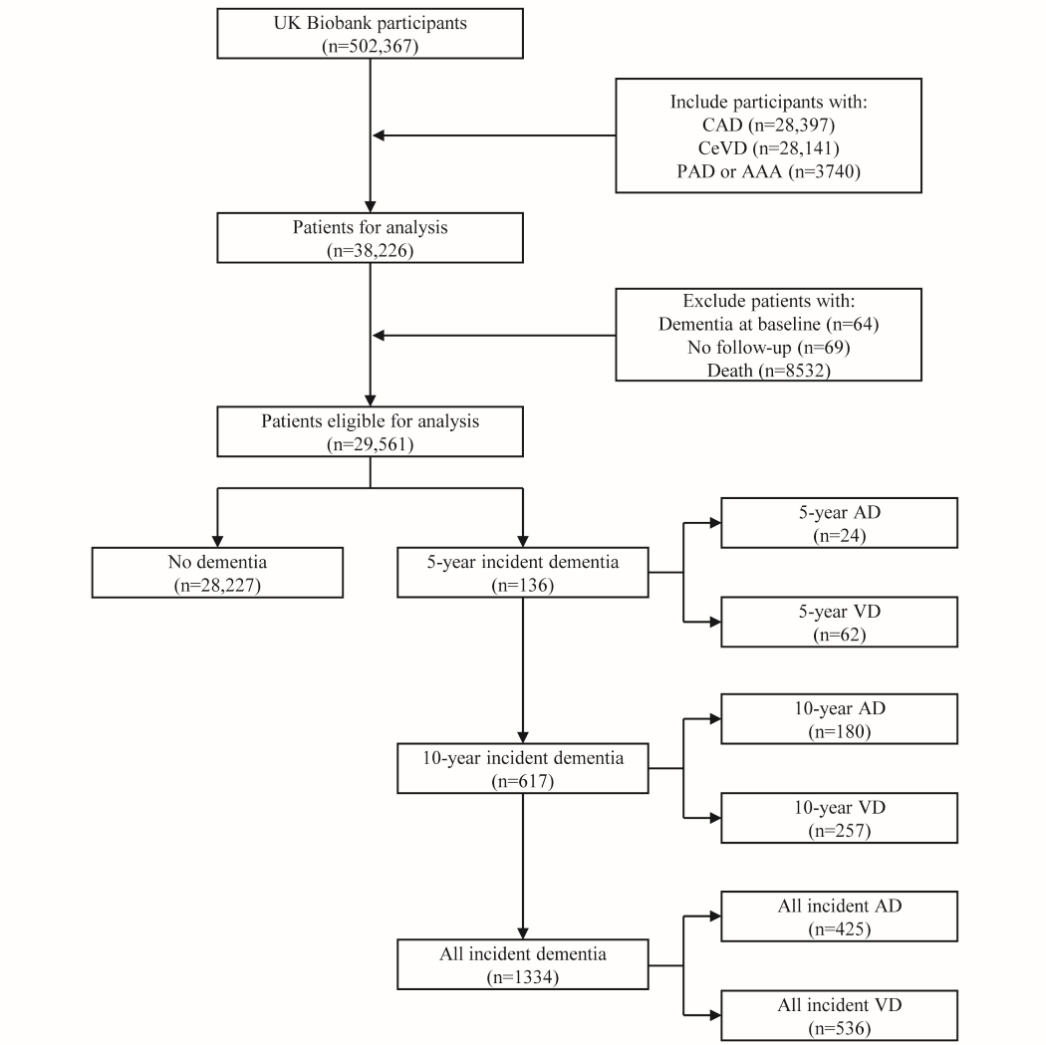
Participant selection flowchart. UK Biobank participants were excluded if baseline dementia was self-reported or follow-up data were absent. The remaining participants were categorized according to their first reported years of dementia, AD, or VD after the baseline. AAA: abdominal aortic aneurysm; AD: Alzheimer disease; CAD: coronary artery disease; CeVD: cerebrovascular disease; PAD: peripheral artery disease; VD: vascular dementia.

We analyzed data from the UK Biobank, a longitudinal prospective study that recruited over 500,000 participants between 2006 and 2010 [16]. The participants were enrolled from 22 recruitment centers across the United Kingdom and were aged between 40 and 69 years at the baseline assessment. Multiple data were collected from the participants, including questionnaires, physical measurements, biological sample assays, genotyping, imaging data, and ongoing hospital records. Figure 1 illustrated the enrollment process, where we included individuals with a prior history of any established ASCVD, such as coronary artery disease (n=28,397), CeVD (n=28,141), peripheral artery disease, or abdominal aortic aneurysm (n=3740) [17]. Participants were excluded at the baseline assessment if they met the following criteria: (1) had dementia at baseline (n=64), (2) had no follow-up records (n=69), and (3) death (n=8532). Ultimately, we included 29,561 participants with ASCVD who had at least 10 years of follow-up until April 2023.

### Ethical Considerations

Ethical approval was obtained from the North West Multi-Centre Research Ethics Committee (11/NW/0382, 16/NW/0274, and 21/NW/0157). Written informed consent was provided by all participants during the collection of primary data. The UK Biobank data used were deidentified, and all personally identifiable information of participants has been removed to ensure privacy and confidentiality. Besides, the UK Biobank offered nonfinancial compensation in the form of travel reimbursements for attending the assessment centers and other incidental expenses related to participation. Additionally, participants were given feedback on their individual health data upon request, which provided valuable insights into their health status. This study adhered to the reporting guidelines of Transparent Reporting of a Multivariable Prediction Model for Individual Prognosis or Diagnosis [18].

### Outcome

The primary end point of this study was the occurrence of all incident dementia, including Alzheimer disease (AD), vascular dementia (VD), frontotemporal dementia, and dementia associated with other neurodegenerative or specified diseases. Due to the high rate of incidence worldwide, AD and VD were examined as the secondary outcomes. To conduct a comprehensive survey on the incidence time, we categorized the patients into 5-year, 10-year, and all incident dementia, AD, and VD. The outcomes were ascertained and categorized based on the International Classification of Diseases and Read codes (Table S1 in Multimedia Appendix 1), which were obtained from the “first occurrence” category in the UK Biobank including the primary care data, the hospital inpatient data, the death register records, and subsequent UK Biobank assessment center visits. Follow-up visits continued until the earliest of the following events: a dementia diagnosis, death, or the most recent available data from either the hospital or the general practitioner, whichever occurred first. What is noteworthy was that the imaging data and lumbar puncture results were not available to doctors to achieve the detailed diagnostic data.

### Data Preparation

In this study, we included all clinically correlated variables during the participants’ baseline visits. The assessment procedure involved a manual examination of each variable to determine its relevance to comprehensively understanding a participant’s overall status. Variables not pertinent to these key domains or lacking in additional insights were excluded. Data screening was processed to exclude noninformative variables with missing values exceeding 40% among all participants. To prevent potential overfitting from oversampling, we applied random undersampling to the majority class, balancing the dataset more effectively. We also adjusted the class weights in our ML algorithms to give more importance to the minority class during model training. Overall, a total of 316 features were adopted, including the participants’ demographic characteristics (n=2), touchscreen-recorded questionnaires (n=151), physical measures (n=66), cognitive function tests (n=22), and biological sample assays (n=59). Furthermore, to improve the informative value of the dataset, we used the available data to generate several variables (n=16) that were not directly extracted from the UK Biobank (Table S2 in Multimedia Appendix 1). Considering the significant impact of ASCVD on mortality, we identified and coded deaths as competing events to ensure accurate modeling of the primary outcome.

In this study, we used different missing data handling strategies tailored to each ML algorithm to ensure the accuracy and robustness of the models. Specifically, for the logistic regression model, we used mean imputation to handle missing values. For each variable with missing data, we calculated its mean in the training dataset and replaced the missing data points with this mean. This method is simple and efficient, making it suitable for models like logistic regression that require complete datasets. For other ML methods, including light gradient boosting machine (LightGBM), extreme gradient boosting machine, random forest, k-nearest neighbor, and artificial neural network, we adopted automatic imputation techniques. These automatic imputation methods leverage the inherent mechanisms of the algorithms or advanced imputation strategies within the preprocessing pipeline to dynamically estimate and replace missing values. To evaluate the robustness of our results, we conducted a sensitivity analysis using multiple imputation by chained equations (Table S4 in Multimedia Appendix 1). This approach generates several imputed datasets by modeling each missing value conditionally based on other variables, thereby accounting for the uncertainty associated with the imputations. By comparing the outcomes across different imputation methods, we assessed the stability and reliability of our predictive models.

To evaluate the model’s stability and generalization across different time periods, this study used a time validation approach to partition dementia data from the UK Biobank database. First, the recruitment date was selected as the primary temporal variable, and all samples were sorted in ascending order based on this date to ensure chronological arrangement and prevent future data from leaking into the training process. Considering previous research and data volume, the dataset was divided into 2 periods: the training and validation sets comprised samples recruited from 2006 to 2009, while the test set included samples diagnosed in 2010. This division ensures that only past data were used for model training, and the model’s predictive performance on future data was assessed during the validation and testing phases. To further guarantee temporal independence, feature selection and standardization were performed exclusively on the training set, with identical transformations applied to the validation and test sets, thereby avoiding the use of information from these sets during training. Given the typically low number of dementia cases, healthy samples with more than 5% missing variables were excluded from the training set to balance the class distribution and enhance model learning. Additionally, multiple imputation methods were used to handle missing data, ensuring data integrity. Through these steps, a time validation framework was established, maintaining the temporal independence and appropriate distribution of the training, validation, and test sets, thereby improving the model’s predictive performance across different time periods and the credibility of the study’s findings.

### Predictor Selection

The predictors for model development were identified through a 2-step process: variable importance ranking and sequential forward selection (SFS) [19,20]. First of all, the importance of each variable was determined using a preliminary trained LightGBM classifier. Gradient boosting machine is a type of boosting that builds these simple models step-by-step, improving the model at each step to better fit the data. LightGBM, developed by Microsoft, is a faster and more efficient version of gradient boosting machine designed to handle large-scale data effectively. The “light” in LightGBM refers to its lightweight nature, meaning it uses less memory and runs faster. The top 50 variables were selected by LightGBM. Next, they were inputted into a hierarchical clustering algorithm, which used Spearman rank-order correlations to further identify and eliminate redundant variables with multicollinearity. We established a correlation threshold of 0.75, considering variables with pairwise correlations above this value as highly redundant. Within each cluster of such variables, we retained only the most predictive variable for the model, effectively reducing multicollinearity while preserving essential information. To avoid overfitting and enhance the robustness of feature selection, a nested cross-validation approach was used. Specifically, in the outer loop, we divided the dataset into multiple folds, selecting 1 fold as the test set and using the remaining folds for feature selection and model training. Within the inner loop, the training set was further split into inner training and validation sets, where features were selected based on performance in the inner validation sets. Finally, model performance was evaluated on the outer test set to ensure fair feature selection and robust predictive capability. Then, an SFS approach was used, wherein the features within the preselected subset underwent reranking according to a newly developed classifier. Afterward, preselected variables were reranked, and multiple ML classifiers were used to sequentially add predictors one at each time. Finally, the classifier was selected based on achieving the best performance of area under the receiver operating characteristic curve (AUC), and we selected the top 10 variables according to the importance of each variable calculated by the LightGBM model. After selecting these 10 variables, adding any other variables did not significantly improve the model. The top 25 predictors are shown in Table 1. More details of predictor selection could be obtained in the part of the Methods section in Multimedia Appendix 1.

**Table 1:** Top 25 predictors for all incident dementia with light gradient boosting machine.

Number	Variables	Importance rating	Ranking
1	Forced vital capacity	0.133	1
2	Summed MET^a^ minutes per week for all activity	0.12	2
3	Age	0.09	3
4	Pairs matching time	0.05	4
5	Mean sphered cell volume	0.038	5
6	Glucose	0.037	6
7	Mean time to correctly identify matches	0.037	7
8	FEV1^b^ *z* score	0.035	8
9	Age first had sexual intercourse	0.033	9
10	C-reactive protein	0.031	10
11	Average weekly red wine intake	0.029	11
12	Calcium	0.029	12
13	Vitamin D	0.028	13
14	Pulse rate automated reading	0.026	14
15	Father age at death	0.025	15
16	Systolic blood pressure automated reading array	0.025	16
17	FEV1/FVC^c^ ratio *z* score	0.024	17
18	Neuroticism score	0.023	18
19	Red blood cell erythrocyte distribution width	0.023	19
20	Apolipoprotein B	0.023	20
21	Total bilirubin	0.023	21
22	Cystatin C	0.022	22
23	Alanine aminotransferase	0.018	23
24	Average weekly beer plus cider intake	0.017	24
25	Result ranking	0.016	25

^a^MET: metabolic equivalent.

^b^FEV1: forced expiratory volume in 1 second.

^c^FVC: forced vital capacity.

### Model Development

We implemented a range of ML techniques, including LightGBM, extreme gradient boosting machine, random forest, logistic regression, k-nearest neighbor, support vector machine, and artificial neural network to classify participants into 2 classes: 0 (predicted to remain no dementia) or class 1 (to develop all incident dementia, AD, or VD). The proposed model was developed using patients with ASCVD without dementia (n=28,227) and with all incident dementia (n=1334) from the UK Biobank dataset. In total, 10 identified predictors were incorporated into the model. We expanded our performance evaluation metrics to include receiver operating characteristic-AUC, precision, recall, and *F*_1_-score, ensuring a comprehensive assessment of the models’ performance on imbalanced data. Subsequently, LightGBM, the best-performing method, was used to develop a dementia risk prediction model of ASCVD, named the UK Biobank ASCVD risk prediction model. The hyperparameter tuning was performed through exhaustive selection from 10,000 sets of candidate parameters, and the optimal set was chosen based on the performance measurement of AUC. Please refer to Table S8 in Multimedia Appendix 1 for detailed information on the search space and final adopted parameters. To evaluate the predictive performance of the models, we constructed and compared the traditional Cox proportional hazards model with the LightGBM model. Both models used identical predictor variables to ensure fairness and consistency in the comparison. The Cox model assessed hazard ratios for each variable through multivariate regression analysis, while the LightGBM model leveraged its robust ability to handle nonlinear relationships and variable interactions for risk prediction. Subsequently, the performance of both models was systematically compared using consistent evaluation metrics (such as AUC) to determine their predictive effectiveness within the study dataset. This comparison aims to validate the potential advantages of the LightGBM model in risk prediction and to provide a reference for the application of the traditional Cox model. The Cox proportional hazards model was also used to account for competing risks, which ensured that the risk of death did not bias the estimation of dementia event probabilities. To enhance the model’s stability and applicability, a time validation approach was used for data analysis. Additionally, we calibrated the raw predicted probabilities into actual dementia risks (Figures S12-S14 in Multimedia Appendix 1). Finally, to assess the clinical utility of the prediction model, decision curve analysis (DCA) was conducted. First, the model’s net benefit was calculated across various threshold probabilities and then compared with the baseline strategies of “treat all” and “treat none.” The DCA curves were plotted using the *rmda* package in R (R Foundation for Statistical Computing) to illustrate the model’s potential value in clinical decision-making. The ML algorithm was implemented using LightGBM library (version 3.3.2) and scikit-learn library (version 1.0.2) in Python (version 3.9; Python Software Foundation).

We also performed a 5-fold cross-validation to assess the stability of feature importance, randomly dividing the dataset into 5 equal parts. In each iteration, 4 folds were used for training, and 1 fold for validation. The training involved 2 stages: model development and calibration. The 4 training folds were split 3:1, with 3 folds for development and 1 fold for calibration. Validation sets were exclusively for performance evaluation. Results were averaged across folds with corresponding SDs.

### Statistical Analysis

In an analysis of the variables of interest, continuous variables were summarized using the median and IQR, while discrete variables were summarized using frequency and percentage. Group comparisons (no dementia vs incident dementia or AD or VD) were conducted using chi-square tests for discrete variables and 2-tailed Student *t* tests for continuous variables. Multivariate analysis was used to calculate odds ratios based on normalized data.

The model’s performance was evaluated using 2 accuracy metrics: discrimination and calibration. Discrimination was assessed using the AUC, which ranges from 0.5 for a noninformative model to 1 for a perfectly discriminating model. Calibration measures the agreement between predicted probabilities and observed event proportions. It was evaluated using the Kolmogorov-Smirnov test with 10 subgroups and visually represented in calibration plots. A *P* value greater than .05 signified an adequate goodness of fit.

Furthermore, we reported accuracy, sensitivity, specificity, precision, and the *F*_1_-score, which were determined using the cutoff that maximized the Youden index. Additionally, we used Shapley Additive Explanations (SHAP) plots to visualize the individual contributions of each predictor to the target variable. All data analysis and visualizations were performed using Python (version 3.9) with packages from the scikit-learn library (version 1.0.2) and the SHAP library (version 0.40.0).

## Results

### Population Characteristics

After quality control, a total of 29,561 participants with ASCVD were included in this study. The median age of the participants was 62.0 (IQR 58.0-66.0) years. Among the participants, 36.63% (10,829/29,561) were women, and 94.12% (27,822/29,561) were White. During a median follow-up time of 10.3 (IQR 7.6-12.4) years, a subset of 1334 participants developed dementia after their baseline visits. Specifically, 617 participants had incidents within 10 years, and 136 had incidents within 5 years. Besides, the prevalence of all-cause dementia was 4.51% (1334/29,561), AD was 1.44% (425/29,561), and VD was 1.81% (536/29,561) in this study. The critical baseline predictors are presented by incident dementia, AD, and VD status in Table 2, and the percentage of missing values for the predictors is shown in Table S3 in Multimedia Appendix 1.

**Table 2:** The baseline characteristics of UK Biobank participants included in the study by dementia, Alzheimer disease (AD), and vascular dementia (VD) status.

Participants characteristics	Overall (n=29,561)	No dementia (n=28,227)	All incident dementia (n=1334)	All incident AD (n=425)	All incident VD (n=536)
Age (years), median (IQR)	62.0 (58.0-66.0)	62.0 (57.0-66.0)	66.0 (63.0-68.0)	66.0 (64.0-68.0)	66.0 (63.0-68.0)
Sex (female), n (%)	10,829 (36.63)	10,353 (36.68)	476 (35.68)	170 (40.00)	163 (30.41)
Ethnicity (White), n (%)	27,822 (94.12)	26,565 (94.11)	1257 (94.23)	404 (95.06)	504 (94.03)
Education (years), median (IQR)	10.0 (9.0-11.0)	10.0 (9.0-11.0)	10.0 (9.0-11.0)	9.0 (9.0-10.0)	9.0 (9.0-10.0)
Forced vital capacity (L), median (IQR)	3.5 (2.9-4.2)	3.5 (2.9-4.2)	3.2 (2.6-3.9)	3.2 (2.7-4.0)	3.1 (2.6-3.9)
Summed MET^a^ minutes per week for all activity (minutes per week), median (IQR)	1662.0 (693.0-3546.0)	1671.0 (698.0-3546.0)	1398.0 (510.0-3288.0)	1653.0 (660.0-3546.0)	1308.0 (408.8-2942.6)
Pairs matching time (seconds), median (IQR)	411.0 (325.0-534.0)	408.0 (324.0-529.0)	487.5 (368.0-678.8)	487.0 (372.0-673.5)	496.0 (370.5-701.0)
Mean sphered cell volume (fL), median (IQR)	82.4 (79.2-85.9)	82.4 (79.2-85.8)	83.1 (79.5-86.7)	82.5 (79.6-86.1)	83.2 (79.2-87.3)
Glucose (mmol/L), median (IQR)	5.0 (4.7-5.5)	5.0 (4.7-5.5)	5.1 (4.7-5.9)	5.1 (4.7-5.6)	5.2 (4.7-6.2)
Mean time to correctly identify matches (seconds), median (IQR)	563.0 (500.0-644.0)	562.0 (500.0-641.0)	594.0 (531.0-699.8)	586.0 (527.0-684.0)	602.0 (532.0-707.5)
FEV1^b^ *z* score (L), median (IQR)	0.6 (–0.1 to 1.3)	0.6 (–0.1 to 1.3)	0.7 (0.0-1.5)	0.5 (–0.0 to 1.4)	0.8 (0.2-1.6)
Age first had sexual intercourse (years), median (IQR)	18.0 (16.0-21.0)	18.0 (16.0-21.0)	18.0 (17.0-21.0)	19.0 (17.0-21.0)	18.0 (16.0-21.0)
C-reactive protein (mg/L), median (IQR)	1.5 (0.8-3.1)	1.5 (0.8-3.1)	1.5 (0.7-3.2)	1.5 (0.7-3.3)	1.7 (0.8-3.4)
CAD^c^, n (%)	21,735 (73.53)	20,763 (73.56)	972 (72.86)	325 (76.47)	357 (66.60)
CeVD^d^, n (%)	21,707 (73.43)	20,738 (73.47)	969 (72.64)	324 (76.24)	356 (66.42)
PAD^e^ or AAA^f^, n (%)	1345 (4.55)	1270 (4.50)	75 (5.62)	23 (5.41)	34 (6.34)

^a^MET: metabolic equivalent.

^b^FEV1: forced expiratory volume in 1 second.

^c^CAD: coronary artery disease.

^d^CeVD: cerebrovascular disease.

^e^PAD: peripheral artery disease.

^f^AAA: abdominal aortic aneurysm.

### Data-Driven Predictors Selection

**Figure 2:**
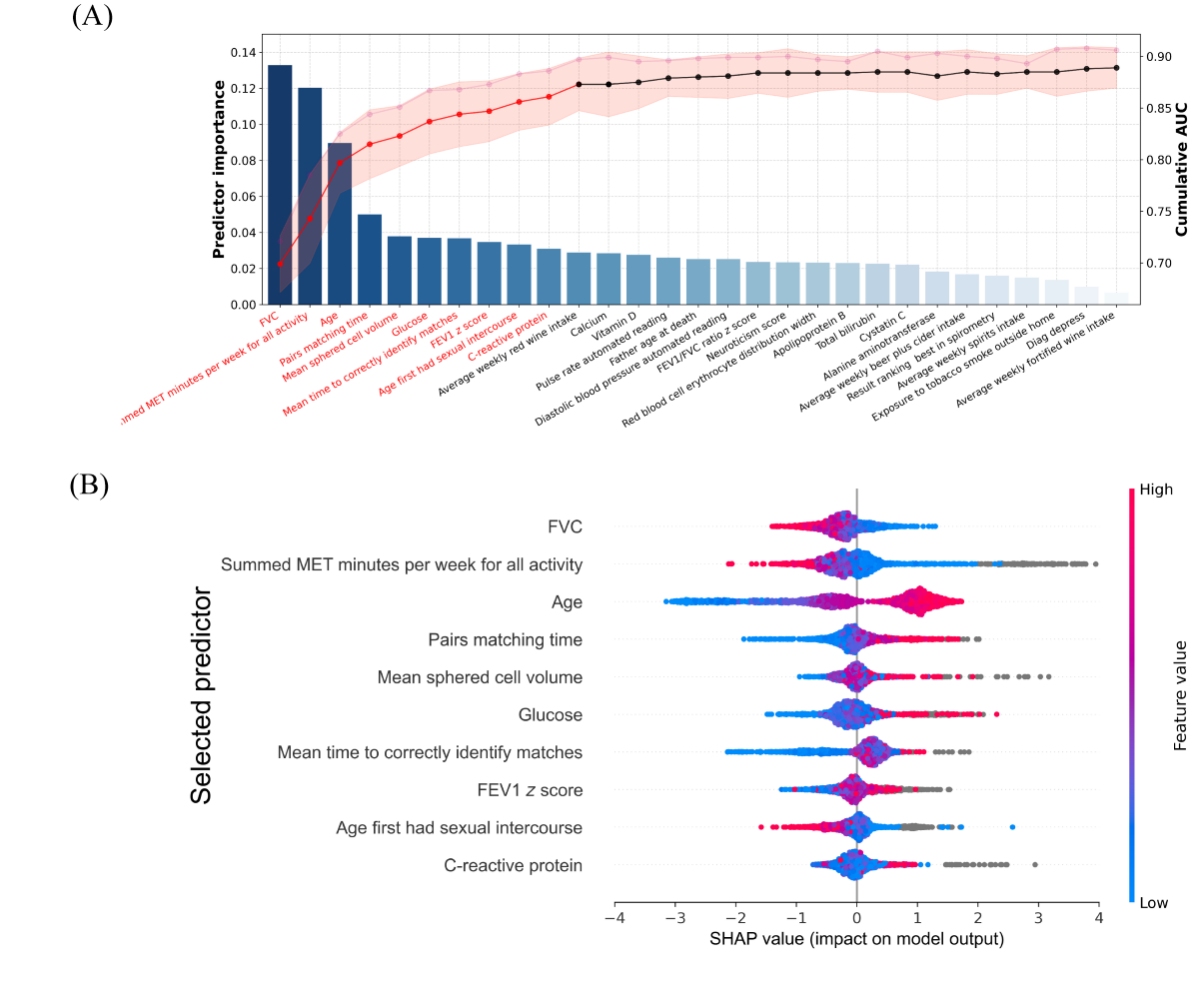
Predictive variable selection and interpretation on all incident dementia. (A) Sequential forward selection from a preselected predictor pool. A bar chart ranked predictor importance by their contribution to classification, while a line chart depicted cumulative AUCs with each iterative predictor inclusion. The top 10 predictors, marked red, were selected for machine learning model construction. (B) SHAP-based visualization of salient predictors. Horizontal bar widths correspond to predictor impact on model predictions, with wider ranges indicating greater influence. Predictor intensity was color-coded, graduating from blue (low) to red (high), as per the color bar on the right. The x-axis orientation signified the probability of either dementia (right) or health (left). AUC: area under the receiver operating characteristic curve; FEV1: forced expiratory volume in 1 second; FVC: forced vital capacity; MET: metabolic equivalent; SHAP: Shapley Additive Explanations.

Among the 316 candidate variables, we initially selected the top 50 variables based on the LightGBM classifier and performed the hierarchical clustering to eliminate the multicollinearity [21]. As shown in the bar chart of Figure 2A, a total of 29 variables were sorted according to their importance in the prediction task. The SFS strategy was used to strike a balance between model performance (AUC on the right axis) and the number of variables selected, as depicted in the line chart. The line chart showed that the model’s performance experienced a sharp increase when incorporating the first few variables and eventually reached a plateau with the inclusion of additional variables. Ultimately, the top 10 variables were chosen as the final predictors for ML model development. Their summary statistics are displayed in Table 2.

### Model Interpretation of Selected Predictors

**Figure 3:**
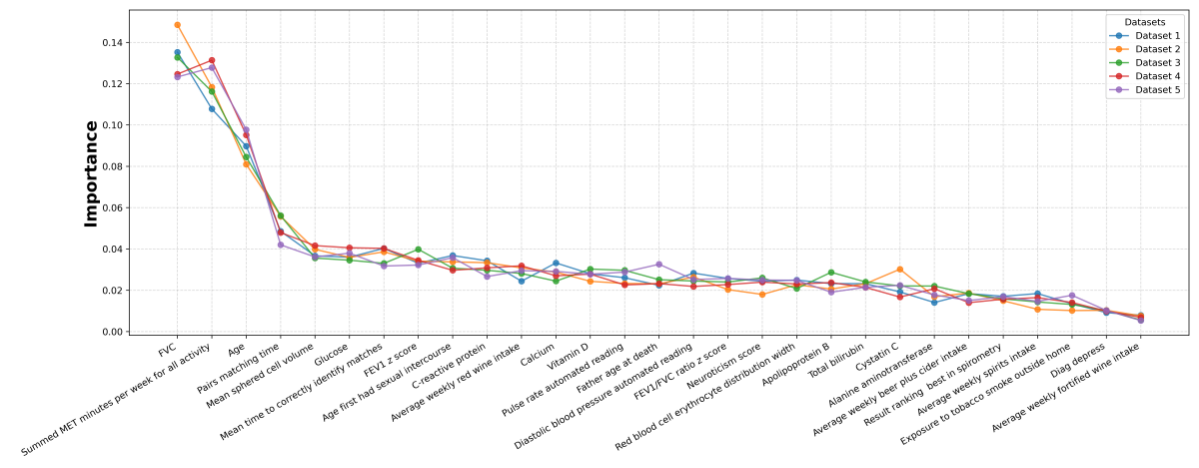
Stability analysis of feature importance across different data subsets in the model of all incident dementia. The stability of feature importance in the model of all incident dementia was assessed across different data subsets, providing insights into the robustness of identified predictors. Each line represented the variability of feature importance in a specific subset, illustrating how consistent the predictive factors are across varying conditions. FEV1: forced expiratory volume in 1 second; FVC: forced vital capacity; MET: metabolic equivalent.

To interpret the influence of each selected predictor, we used SHAP values and visualized them in Figure 2B. The predictors were interpreted based on value magnitude (coded by gradient colors) and tendency on the horizontal axis (indicting the likelihood of developing dementia). Take the predictor forced vital capacity (FVC) as an example. Patients with ASCVD with lower FVC values (colored blue) were more likely to develop dementia (right side) compared to those with higher FVC values (colored red). Similarly, for the remaining predictors, patients with ASCVD who spend less time engaging in activities, being older, who take longer time to complete pair matching tasks, and who have higher mean sphered cell volume (MSCV), forced expiratory volume in 1 second *z* score, C-reactive protein, and glucose levels tend to have an increased risk of developing dementia. Interestingly, we found that patients who engaged in sexual intercourse at an earlier age were more likely to develop dementia. Moreover, a 5-fold cross-validation stability analysis of feature importance was conducted. The results indicated that most features exhibited high stability across different data subsets. Figure 3 shows the distribution of key feature importance across all folds.

### Model Performance Across Different Populations and Algorithms

Compared with other ML algorithms, it can be seen in Table 3 that LightGBM demonstrated superior performance across various metrics. We used the AUC metric to evaluate the discrimination performance of the UK Biobank ASCVD risk prediction model. As depicted in Table 4, the model achieved a mean AUC of 0.866 (SD 0.027) for all incident dementia cases. Furthermore, the model demonstrated promising results for the prediction of 10-year and 5-year incident dementia, with mean AUCs of 0.876 (SD 0.024) and 0.903 (SD 0.076), respectively. The model for all incident dementia exhibited a mean accuracy of 0.883 (SD 0.01), mean sensitivity of 0.637 (SD 0.084), mean specificity of 0.914 (SD 0.012), mean precision of 0.479 (SD 0.031), and mean *F*_1_-score of 0.546 (SD 0.043). Apart from the 5-year AD and VD predictions, the model also displayed valuable discrimination abilities for different AD and VD population groups. Specifically, the mean AUCs for all and 10-year incident AD were 0.836 (SD 0.043) and 0.828 (SD 0.112), respectively, while the mean AUCs for all and 10-year incident VD achieved 0.870 (SD 0.029) and 0.881 (SD 0.031), respectively. For specific metrics of all, 10-year, and 5-year dementia, AD, and VD predictions, please refer to Table 4 and Figures S3-S11 and S15 in Multimedia Appendix 1. We also compared the performance of the traditional Cox proportional hazards model and the LightGBM-based model in risk prediction. The AUC of all incident dementia, AD, and VD for the Cox model were 0.67, 0.67, and 0.71, respectively (Figure S1 in Multimedia Appendix 1). After competing risk analysis with death, the prediction power of the Cox model did not show a significant difference in predicting AD and VD (Tables S5-S7 and Figure S2 in Multimedia Appendix 1).

**Table 3:** Model performance metrics for different machine learning classifiers on all incident dementiaa.

	Accuracy, mean (SD)	Sensitivity, mean (SD)	Specificity, mean (SD)	Precision, mean (SD)	*F*_1_-score, mean (SD)	AUC^b^, mean (SD)
LightGBM^c^	0.883 (0.010)	0.637 (0.084)	0.914 (0.012)	0.479 (0.031)	0.546 (0.043)	0.866 (0.027)
XGBoost^d^	0.814 (0.01)	0.709 (0.045)	0.826 (0.01)	0.323 (0.018)	0.444 (0.024)	0.853 (0.02)
Random forest	0.829 (0.01)	0.703 (0.047)	0.844 (0.012)	0.345 (0.02)	0.463 (0.024)	0.859 (0.02)
KNN^e^	0.829 (0.014)	0.561 (0.059)	0.86 (0.012)	0.32 (0.033)	0.407 (0.041)	0.765 (0.024)
Logistic regression	0.783 (0.014)	0.627 (0.071)	0.802 (0.016)	0.27 (0.022)	0.378 (0.031)	0.795 (0.035)
ANN^f^	0.836 (0.1)	0.638 (0.145)	0.859 (0.125)	0.377 (0.174)	0.46 (0.102)	0.833 (0.025)

^a^The cutoff for binarization was established by maximizing the Youden index (YI=sensitivity+specificity–1).

^b^AUC: area under the receiver operating characteristic curve.

^c^LightGBM: light gradient boosting machine.

^d^XGBoost: extreme gradient boosting machine.

^e^KNN: k-nearest neighbor.

^f^ANN: artificial neural network.

**Table 4:** Model performance metrics for the prediction on different types of dementia^a^.

	Accuracy, mean (SD)	Sensitivity, mean (SD)	Specificity, mean (SD)	Precision, mean (SD)	*F*_1_-score, mean (SD)	AUC^b^, mean (SD)
All incident dementia	0.883 (0.01)	0.637 (0.084)	0.914 (0.012)	0.479 (0.031)	0.546 (0.043)	0.866 (0.027)
All incident AD^c^	0.847 (0.029)	0.656 (0.125)	0.855 (0.035)	0.148 (0.01)	0.241 (0.012)	0.836 (0.043)
All incident VD^d^	0.859 (0.039)	0.683 (0.045)	0.868 (0.043)	0.206 (0.045)	0.315 (0.053)	0.870 (0.029)
10-Year incident dementia	0.877 (0.033)	0.709 (0.059)	0.886 (0.037)	0.255 (0.057)	0.374 (0.063)	0.876 (0.024)
10-Year incident AD	0.668 (0.061)	0.814 (0.214)	0.666 (0.065)	0.036 (0.006)	0.07 (0.012)	0.828 (0.112)
10-Year incident VD	0.839 (0.051)	0.757 (0.1)	0.841 (0.055)	0.098 (0.02)	0.173 (0.029)	0.881 (0.031)
5-Year incident dementia	0.939 (0.031)	0.694 (0.155)	0.942 (0.033)	0.125 (0.045)	0.211 (0.069)	0.903 (0.076)
5-Year incident AD	0.979 (0.006)	0.4 (0.5)	0.98 (0.006)	0.039 (0.049)	0.061 (0.047)	0.775 (0.243)
5-Year incident VD	0.952 (0.012)	0.471 (0.288)	0.957 (0.012)	0.106 (0.063)	0.172 (0.102)	0.803 (0.11)

^a^Cutoffs were established by maximizing the Youden index (YI=sensitivity+specificity–1).

^b^AUC: area under the receiver operating characteristic curve.

^c^AD: Alzheimer disease.

^d^VD: vascular dementia.

**Figure 4:**
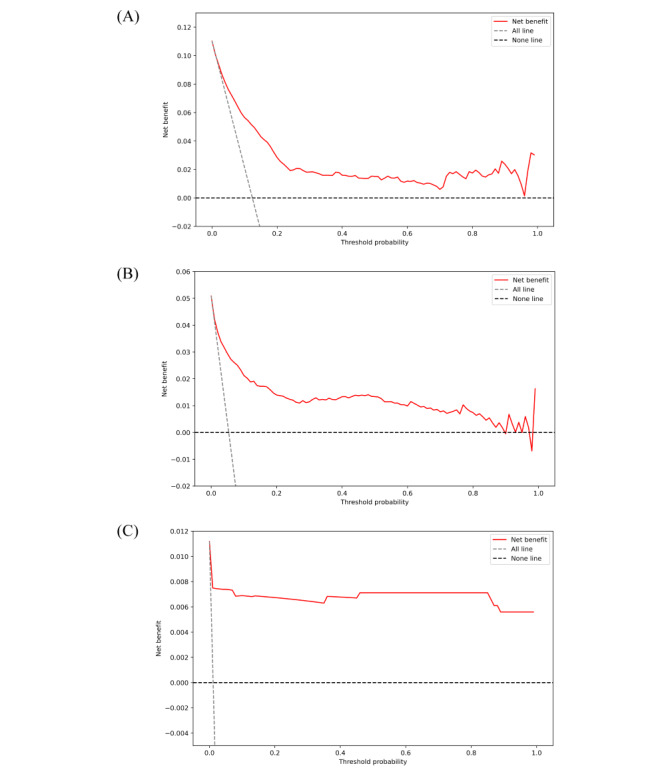
Clinical applicability of dementia risk prediction with a decision curve analysis. (A-C) The decision curve analysis of the UK Biobank atherosclerotic cardiovascular disease risk prediction model on all incident and 10-year incident and 5-year incident times.

DCA demonstrated that our prediction model exhibited a higher net benefit within the threshold probability range of 0.04 to 0.97 across different time periods, significantly outperforming both the “treat all” and “treat none” baseline strategies (Figure 4). The Kolmogorov-Smirnov test was conducted to assess the calibration of the model. A *P* value greater than .05 indicates sufficient goodness of fit. Satisfactory calibrations for the development of all population groups, including 5-year or 10-year or all incident dementia, AD, and VD, were observed (Table 5 and Figures S12-S14 in Multimedia Appendix 1).

**Table 5:** Calibration data of the UK Biobank atherosclerotic cardiovascular disease risk prediction model^a^.

Decile groups (10% quantile each)	All incident dementia^b^		All incident AD^b,c^			All incident VD^b,d^	
	Observed probability (‰)	Preserved probability (‰)	Observed probability (‰)	Preserved probability (‰)	Observed probability (‰)		Preserved probability (‰)
1	4.71	3.53	2.83	2.10	1.79		1.45
2	9.40	11.72	2.17	5.46	0.98		4.60
3	24.78	21.98	15.58	8.93	8.03		8.37
4	35.91	33.55	12.88	12.13	10.98		12.72
5	41.65	45.96	14.84	15.23	17.91		17.39
6	53.92	59.90	18.47	18.97	22.79		22.36
7	77.65	77.23	19.95	23.72	30.75		28.81
8	96.51	101.12	28.01	30.43	35.79		37.58
9	132.95	141.49	42.09	43.31	47.42		52.26
10	627.36	606.99	213.22	209.58	293.58		283.14

^a^Calibration data of the UK Biobank atherosclerotic cardiovascular disease risk prediction model on different types of dementia at all incident times. The 5-fold cross-validation strategy was performed to calculate the results. A *P* value less than .05 indicated the statistical significance of the results.

^b^Goodness-of-fit *P* value >.99.

^c^AD: Alzheimer disease.

^d^VD: vascular dementia.

### A Temporal Validation of the Constructed Model

To assess the stability and generalizability of our dementia prediction model, we used a time validation approach using data from the UK Biobank. The model was trained and validated on samples recruited between 2006 and 2009 and tested on the 2010 cohort. In the training and validation set, the model achieved a mean AUC of 0.866 (SD 0.027) in predicting all incident dementia, indicating strong discriminatory ability. When applied to the test set from 2010, the model maintained robust performance with an AUC of 0.819, suggesting good generalization to future data. Other performance metrics in the test set: accuracy was 0.851, sensitivity was 0.691, specificity was 0.866, precision was 0.315, and the *F*_1_-score was 0.433 (Table 6).

**Table 6:** Model performance metrics for the prediction on the test data divided by time period^a^.

	Accuracy	Sensitivity	Specificity	Precision	*F*_1_-score	AUC^b^
All incident dementia	0.851	0.691	0.866	0.315	0.433	0.819
All incident AD^c^	0.921	0.500	0.933	0.182	0.267	0.718
All incident VD^d^	0.916	0.619	0.926	0.218	0.322	0.838
10-Year incident dementia	0.925	0.576	0.943	0.350	0.435	0.824
10-Year incident AD	0.916	0.517	0.922	0.093	0.157	0.804
10-Year incident VD	0.946	0.590	0.953	0.207	0.307	0.834
5-Year incident dementia	0.984	0.480	0.991	0.4	0.436	0.882
5-Year incident AD	0.181	1	0.181	0.001	0.002	0.605
5-Year incident VD	0.002	1	0	0.002	0.004	0.595

^a^Cutoffs were established by maximizing the Youden index (YI=sensitivity+specificity–1).

^b^AUC: area under the receiver operating characteristic curve.

^c^AD: Alzheimer disease.

^d^VD: vascular dementia.

## Discussion

### Principal Findings and Comparisons With Prior Work

In this study, we developed a predictive model using the LightGBM algorithm and leveraging big data from the UK Biobank to assess the risk of dementia in patients with ASCVD. To the best of our knowledge, this is the first model that uses big data to predict the risk of dementia specifically in patients with ASCVD. Our model incorporates 10 clinical predictive factors, selected based on their importance, to accurately estimate the risk of dementia. Notably, our model demonstrates particularly strong performance in predicting all-cause dementia and VD, with AUC values exceeding 0.85. Furthermore, the model effectively calibrates the predicted probabilities and aligns well with the observed event ratios, indicating its reliability and accuracy in estimating the risk of dementia in patients with ASCVD.

The ASCVD has long been recognized as one of the most significant risk factors for dementia, especially VD [22]. Although previous studies have primarily concentrated on the influence of atherosclerotic CeVDs on dementia, emerging research suggests that systemic atherosclerotic diseases beyond CeVDs also significantly contribute to the development of dementia [23]. To the best of our knowledge, no prior studies have used big data to predict the risk of dementia in patients with ASCVD. Unlike models based on variables obtained from intricate neuroimaging and neuropsychological tests, the predictors in this model are more accessible and can be applied in various clinical settings and medical institutions.

Recent studies have indicated the importance of vascular risk factors in the development of dementia, which should be taken into consideration by clinical practitioners. During the establishment of a dementia risk model in the population with ASCVD, we identified several key and distinctive risk factors that differ from other longitudinal studies. Age was identified as one of the most significant influencing factors in this study. Other factors include low exercise time, high fasting blood glucose, and reaction time including pair matching time and mean time to correctly identify matches. These risk factors have been shown to have a close relationship with the overall health of the vascular system and are critical in the development of ASCVD events [24,25]. Besides, high plasma levels of C-reactive protein at baseline were associated with a high risk of all incident dementia in this study, which is corresponded with the result of the latest research [26]. Furthermore, we found that MSCV should be considered as a new and significant factor in assessing the risk of dementia in patients with ASCVD. MSCV is primarily a parameter in hematology used to assess changes in the volume of spherocytes. Currently, there is no evidence to suggest a direct relationship between MSCV and dementia. However, overall blood health can indirectly affect cognitive function, especially in the presence of chronic anemia or other systemic diseases [27]. If an abnormal MSCV is observed in clinical practice or research, it is important to consider the patient’s overall health status comprehensively, including but not limited to neurological function, to fully evaluate the patient’s dementia risk. Recent studies have also gradually found that lung function may play a role in the onset of dementia by influencing brain structure [28]. Our model indicated FVC and forced expiratory volume in 1 second *z* score as protective factors in decreasing dementia risk. Finally, age first had sexual intercourse, also known as age at first intercourse (AFS), was first identified as one of the significant risk factors for dementia in populations with ASCVD, which is interesting and worth attention. Compared to other biological traits, reproductive behaviors, especially sexual factors, have long been neglected when it comes to the study of CVDs and neurological disorders. Recent studies show that the earlier the age of first sexual intercourse, the higher the likelihood of developing hypertension and CVDs [29,30]. The specific processes driving this relationship are not yet fully understood, but they may include a mix of environmental and genetic influences. For instance, early sexual activities are often accompanied by adverse environmental factors, including lower educational attainment, increased smoking and alcohol consumption, and the use of illicit drugs [31], which are all closely associated with CVDs. Recent studies have identified a causal relationship between AFS and CVDs at the genetic level [30,32]. Although AFS has no direct impact on dementia, it might be induced from our study that AFS is indirectly related to dementia through intriguing CVDs especially in patients with ASCVD.

In our study, we performed a stability analysis of feature importance using 5-fold cross-validation to ensure the robustness of the identified predictors. The analysis revealed that several key features consistently ranked highly across all folds, indicating their strong and reliable association with the outcome. Specifically, FVC, summed metabolic equivalent minutes per week, age, and pair matching time maintained relatively high importance scores in every subset, underscoring their role as robust predictors. Although these 4 variables exhibited significant variation compared to other variables, the impact of this variation is minimal relative to their importance.

We acknowledge that the Cox proportional hazards model, as a mature and interpretable method, holds a significant position in survival analysis. However, our study results show that the LightGBM-based ML model significantly outperforms the Cox model in predictive performance metrics such as AUC, demonstrating its advantages in handling nonlinear relationships and complex interactions between variables. LightGBM effectively captures patterns in high-dimensional data, thereby enhancing the accuracy of risk prediction. Although ML models face certain challenges in terms of computational resources and interpretability, their substantial improvement in predictive performance illustrates their added value in practical applications. Future research could explore combining traditional Cox models with ML methods to balance predictive performance and model interpretability, thereby meeting diverse clinical application needs. Besides, our study indicated that mortality had a minimal impact on the primary outcomes, and the overall conclusions of the study remained largely unchanged. This suggested that the prediction power of our model remained robust even after accounting for competing risks.

The time validation results demonstrate that our dementia prediction model maintains robust performance across different temporal cohorts. Specifically, in the population of all incident dementia, the AUC remained consistently high, with a slight decrease from 0.866 in the training set to 0.819 in the test set. Similarly, other performance metrics such as accuracy, precision, recall, and *F*_1_-score showed only minor declines over time. This stability suggests that the model effectively captures underlying patterns associated with dementia risk that are persistent across the studied time periods. The consistency of performance metrics across the training and test sets indicates that the model’s predictive capabilities are not significantly affected by temporal shifts in the data. The stable performance of the model over different time periods enhances its long-term applicability in clinical and public health settings. A model that maintains its predictive accuracy over time is invaluable for ongoing and future dementia screening programs, enabling early identification of at-risk individuals with confidence in its sustained reliability. However, it is also essential to acknowledge the significant performance decline observed in the test set when predicting the risk of 5-year AD and VD, which may be attributed to the low prevalence in patients with ASCVD during a relative short period. To ensure continued efficacy, periodic retraining and validation of the model with new data may be necessary. This approach would allow the model to adapt to any emerging trends or shifts in risk factors that may influence dementia incidence over time.

In clinical practice, the LightGBM model can be applied during the initial diagnosis or follow-up stages to early identify individuals with high dementia risk among patients with ASCVD, thus promoting timely intervention and treatment. For instance, using this predictive model during a patient’s initial visit can assist physicians in swiftly identifying patients at high risk and arranging further diagnostic tests or interventions. Moreover, integrating the model’s predictions into electronic health record systems can generate alerts and recommend further evaluations, thereby enhancing diagnostic accuracy and personalized treatment plans. To effectively communicate the predicted risk, doctors should use easily understandable language to explain the model results and their implications to the patient while also providing clear next steps and support resources to alleviate patient anxiety. Consider a hypothetical example: a 55-year-old male patient with hypertension and high cholesterol who recently experienced a heart attack. After using the LightGBM model for assessment, the results indicate a high dementia risk. Based on this, the doctor decides to schedule detailed cognitive function tests and recommends a comprehensive plan that includes cognitive training, a healthy diet, and regular exercise. Through these interventions, the patient can better manage his cardiovascular health while taking steps to reduce the likelihood of developing dementia.

However, the application of such predictive models raises potential ethical issues. First, there may be prediction bias due to training data, leading to unequal care, so continuous monitoring and validation are needed to ensure fairness. Second, doctors need to carefully communicate the model’s predictions to avoid causing unnecessary anxiety for patients. Additionally, patient data use should require clear consent and ensure privacy protection. Finally, caution against overreliance on model predictions is necessary, with doctors maintaining primary responsibility for care decisions.

### Limitations

Several limitations should be considered when interpreting the results. First, our study focused on a specific population of patients with ASCVD. Due to the relatively small sample size, we observed a lower AUC value when predicting the 5-year incidence rates of dementia, especially for AD and VD incidence over a 5-year period. This issue can be addressed by further expanding the sample size. Additionally, this study primarily used samples of European descent, which may restrict the generalizability of our findings to other populations. Genetic, environmental, and lifestyle differences across diverse ethnic groups could influence the model’s performance and predictive accuracy. The limited diversity of the sample may affect the model’s applicability to non-European populations. To ensure broader relevance and robustness, future research should include diverse ethnic backgrounds to validate and potentially refine the model for varied demographic groups. While the time validation results are promising, the model currently relies on static features collected at baseline. Integrating longitudinal data and time-varying covariates could potentially improve predictive performance and adaptability over extended periods. Despite incorporating death as a primary competing risk, there might still be other unrecognized or unadjusted competing factors, such as other chronic diseases or lifestyle changes, that could influence the results to some extent. The application of competing risk models relied on the correct specification of models and assumptions; any biases in model setup might affect the accuracy of the analysis. Therefore, future research should further explore additional potential competing risk factors and use more sophisticated statistical methods to comprehensively assess the prediction power. Regarding the ML algorithms chosen for our study, the use of LightGBM may lead to data overfitting because it generates deep decision trees. To mitigate overfitting, a maximum depth limit should be imposed during the use of LightGBM. Furthermore, it is important to acknowledge that LightGBM is a bias-based algorithm and can be sensitive to noise in data processing, which may potentially affect the final data analysis results. Additionally, it should be noted that the predictive variables identified in this study were derived from data-driven analytical models, which may induce some bias compared to actual clinical diagnostic and treatment experiences. While advanced predictive models and results have been obtained, their applicability to clinical practice remains uncertain. Therefore, future research should focus on validating the analysis results using other independent cohorts with larger sample sizes and extending the study methodology to populations from different countries, regions, and ethnicities. The integration of clinical practice experiences will contribute to the development of more universally applicable and practical models.

### Conclusions

This study has identified several practical and novel predictors for dementia screening in patients with ASCVD. It is worthy of testing and evaluating the applicability of these factors in clinical practice. Future studies should focus on investigating whether intervening in these factors can help prevent the incidence of dementia in patients with ASCVD. By exploring these possibilities, we can potentially improve the management and outcomes of patients with ASCVD and reduce the burden of dementia in this population.

## Appendix

Multimedia Appendix 1 UK Biobank atherosclerotic cardiovascular disease study supplementary tables, figures, and methodological details.

## Abbreviations

AD: Alzheimer disease
AFS: age at first intercourse
ASCVD: atherosclerotic cardiovascular disease
AUC: area under the receiver operating characteristic curve
CeVD: cerebrovascular disease
CVD: cardiovascular disease
DCA: decision curve analysis
FVC: forced vital capacity
LightGBM: light gradient boosting machine
ML: machine learning
MSCV: mean sphered cell volume
SFS: sequential forward selection
SHAP: Shapley Additive Explanations
VD: vascular dementia

## Data Availability

All data used during this study are available from the UK Biobank Resource. As the datasets are the property of UK Biobank, they are not available for direct request. Researchers interested in the data can apply for access through the UK Biobank Access Management System.

## References

[1] Roth GA, Mensah GA, Johnson CO, Addolorato G, Ammirati E, Baddour LM, Barengo NC, Beaton AZ, Benjamin EJ, Benziger CP, Bonny A, Brauer M, Brodmann M, Cahill TJ, Carapetis J, Catapano AL, Chugh SS, Cooper LT, Coresh J, Criqui M, DeCleene N, Eagle KA, Emmons-Bell S, Feigin VL, Fernández-Solà J, Fowkes G, Gakidou E, Grundy SM, He FJ, Howard G, Hu F, Inker L, Karthikeyan G, Kassebaum N, Koroshetz W, Lavie C, Lloyd-Jones D, Lu HS, Mirijello A, Temesgen AM, Mokdad A, Moran AE, Muntner P, Narula J, Neal B, Ntsekhe M, Moraes de Oliveira G, Otto C, Owolabi M, Pratt M, Rajagopalan S, Reitsma M, Ribeiro ALP, Rigotti N, Rodgers A, Sable C, Shakil S, Sliwa-Hahnle K, Stark B, Sundström J, Timpel P, Tleyjeh IM, Valgimigli M, Vos T, Whelton PK, Yacoub M, Zuhlke L, Murray C, Fuster V (2020). Global burden of cardiovascular diseases and risk factors, 1990-2019: update from the GBD 2019 study. J Am Coll Cardiol.

[2] GBD 2017 Causes of Death Collaborators (2018). Global, regional, and national age-sex-specific mortality for 282 causes of death in 195 countries and territories, 1980-2017: a systematic analysis for the Global Burden of Disease Study 2017. Lancet.

[3] Zhao D, Liu J, Wang M, Zhang X, Zhou M (2019). Epidemiology of cardiovascular disease in China: current features and implications. Nat Rev Cardiol.

[4] Livingston G, Huntley J, Sommerlad A, Ames D, Ballard C, Banerjee S, Brayne C, Burns A, Cohen-Mansfield J, Cooper C, Costafreda SG, Dias A, Fox N, Gitlin LN, Howard R, Kales HC, Kivimäki M, Larson EB, Ogunniyi A, Orgeta V, Ritchie K, Rockwood K, Sampson EL, Samus Q, Schneider LS, Selbæk Geir, Teri L, Mukadam N (2020). Dementia prevention, intervention, and care: 2020 report of the Lancet Commission. Lancet.

[5] Winstein CJ, Stein J, Arena R, Bates B, Cherney LR, Cramer SC, Deruyter F, Eng JJ, Fisher B, Harvey RL, Lang CE, MacKay-Lyons M, Ottenbacher KJ, Pugh S, Reeves MJ, Richards LG, Stiers W, Zorowitz RD (2016). Guidelines for adult stroke rehabilitation and recovery: a guideline for healthcare professionals from the American Heart Association/American Stroke Association. Stroke.

[6] Sabia S, Dugravot A, Dartigues JF, Abell J, Elbaz A, Kivimäki M, Singh-Manoux A (2017). Physical activity, cognitive decline, and risk of dementia: 28 year follow-up of Whitehall II cohort study. BMJ.

[7] Sabia S, Fayosse A, Dumurgier J, Schnitzler A, Empana J, Ebmeier KP, Dugravot A, Kivimäki M, Singh-Manoux A (2019). Association of ideal cardiovascular health at age 50 with incidence of dementia: 25 year follow-up of Whitehall II cohort study. BMJ.

[8] Schwarzinger M, Pollock BG, Hasan OSM, Dufouil C, Rehm J, QalyDays Study Group (2018). Contribution of alcohol use disorders to the burden of dementia in France 2008-13: a nationwide retrospective cohort study. Lancet Public Health.

[9] Veronese N, Facchini S, Stubbs B, Luchini C, Solmi M, Manzato E, Sergi G, Maggi S, Cosco T, Fontana L (2017). Weight loss is associated with improvements in cognitive function among overweight and obese people: a systematic review and meta-analysis. Neurosci Biobehav Rev.

[10] Nordestgaard LT, Christoffersen M, Frikke-Schmidt R (2022). Shared risk factors between dementia and atherosclerotic cardiovascular disease. Int J Mol Sci.

[11] Deo RC (2015). Machine learning in medicine. Circulation.

[12] James C, Ranson JM, Everson R, Llewellyn DJ (2021). Performance of machine learning algorithms for predicting progression to dementia in memory clinic patients. JAMA Netw Open.

[13] Zhan Y, Chen K, Wu X, Zhang D, Zhang J, Yao L, Guo X (2015). Identification of conversion from normal elderly cognition to Alzheimer's disease using multimodal support vector machine. J Alzheimers Dis.

[14] Park JH, Cho HE, Kim JH, Wall MM, Stern Y, Lim H, Yoo S, Kim HS, Cha J (2020). Machine learning prediction of incidence of Alzheimer's disease using large-scale administrative health data. NPJ Digit Med.

[15] Cui Y, Liu B, Luo S, Zhen X, Fan M, Liu T, Zhu W, Park M, Jiang T, Jin JS (2011). Identification of conversion from mild cognitive impairment to Alzheimer's disease using multivariate predictors. PLoS One.

[16] Sudlow C, Gallacher J, Allen N, Beral V, Burton P, Danesh J, Downey P, Elliott P, Green J, Landray M, Liu B, Matthews P, Ong G, Pell J, Silman A, Young A, Sprosen T, Peakman T, Collins R (2015). UK Biobank: an open access resource for identifying the causes of a wide range of complex diseases of middle and old age. PLoS Med.

[17] Hageman SHJ, McKay AJ, Ueda P, Gunn LH, Jernberg T, Hagström E, Bhatt DL, Steg PG, Läll K, Mägi R, Gynnild MN, Ellekjær H, Saltvedt I, Tuñón J, Mahíllo I, Aceña Á, Kaminski K, Chlabicz M, Sawicka E, Tillman T, McEvoy JW, Di Angelantonio E, Graham I, De Bacquer D, Ray KK, Dorresteijn JAN, Visseren FLJ (2022). Estimation of recurrent atherosclerotic cardiovascular event risk in patients with established cardiovascular disease: the updated SMART2 algorithm. Eur Heart J.

[18] Collins GS, Reitsma JB, Altman DG, Moons KG (2015). Transparent Reporting of a multivariable prediction model for Individual Prognosis Or Diagnosis (TRIPOD): the TRIPOD statement. BMJ.

[19] Wang J, Qiu J, Zhu T, Zeng Y, Yang H, Shang Y, Yin J, Sun Y, Qu Y, Valdimarsdóttir UA, Song H (2023). Prediction of suicidal behaviors in the middle-aged population: machine learning analyses of UK Biobank. JMIR Public Health Surveill.

[20] Li Q, Yang X, Xu J, Guo Y, He X, Hu H, Lyu T, Marra D, Miller A, Smith G, DeKosky S, Boyce RD, Schliep K, Shenkman E, Maraganore D, Wu Y, Bian J (2023). Early prediction of Alzheimer's disease and related dementias using real-world electronic health records. Alzheimers Dement.

[21] Martin SA, Townend FJ, Barkhof F, Cole JH (2023). Interpretable machine learning for dementia: a systematic review. Alzheimers Dement.

[22] Iadecola C (2020). Revisiting atherosclerosis and dementia. Nat Neurosci.

[23] Zlokovic BV, Gottesman RF, Bernstein KE, Seshadri S, McKee A, Snyder H, Greenberg SM, Yaffe K, Schaffer CB, Yuan C, Hughes TM, Daemen MJ, Williamson JD, González HM, Schneider J, Wellington CL, Katusic ZS, Stoeckel L, Koenig JI, Corriveau RA, Fine L, Galis ZS, Reis J, Wright JD, Chen J (2020). Vascular contributions to cognitive impairment and dementia (VCID): a report from the 2018 National Heart, Lung, and Blood Institute and National Institute of Neurological Disorders and Stroke Workshop. Alzheimers Dement.

[24] Meyer-Lindemann U, Moggio A, Dutsch A, Kessler T, Sager HB (2023). The impact of exercise on immunity, metabolism, and atherosclerosis. Int J Mol Sci.

[25] Stone NJ, Smith SC, Orringer CE, Rigotti NA, Navar AM, Khan SS, Jones DW, Goldberg R, Mora S, Blaha M, Pencina MJ, Grundy SM (2022). Managing atherosclerotic cardiovascular risk in young adults: JACC state-of-the-art review. J Am Coll Cardiol.

[26] Tachibana A, Iga JI, Ozaki T, Yoshida T, Yoshino Y, Shimizu H, Mori T, Furuta Y, Shibata M, Ohara T, Hata J, Taki Y, Mikami T, Maeda T, Ono K, Mimura M, Nakashima K, Takebayashi M, Ninomiya T, Ueno S (2024). Serum high-sensitivity C-reactive protein and dementia in a community-dwelling Japanese older population (JPSC-AD). Sci Rep.

[27] Qiang YX, Deng YT, Zhang YR, Wang H, Zhang W, Dong Q, Feng J, Cheng W, Yu J (2023). Associations of blood cell indices and anemia with risk of incident dementia: a prospective cohort study of 313,448 participants. Alzheimers Dement.

[28] Ma YH, Shen LX, Li YZ, Leng Y, Yang L, Chen S, He X, Zhang Y, Chen R, Feng J, Tan L, Dong Q, Suckling J, David Smith A, Cheng W, Yu J (2023). Lung function and risk of incident dementia: a prospective cohort study of 431,834 individuals. Brain Behav Immun.

[29] Ngueta G, Ndjaboue R (2018). Early sexual experience and hypertension in US adults: results from the National Health and Nutrition Examination Survey 2001-2016. J Hypertens.

[30] Zhuo C, Chen L, Wang Q, Cai H, Lin Z, Pan H, Wu M, Jin Y, Jin H, Zheng L (2023). Association of age at first sexual intercourse and lifetime number of sexual partners with cardiovascular diseases: a bi-directional Mendelian randomization study. Front Cardiovasc Med.

[31] Boisvert I, Boislard MA, Poulin F (2017). Early sexual onset and alcohol use and misuse from adolescence into young adulthood. J Adolesc Health.

[32] Mills MC, Tropf FC, Brazel DM, van Zuydam N, Vaez A, Pers TH, Snieder H, Perry JRB, Ong KK, den Hoed M, Barban N, Day FR (2021). Identification of 371 genetic variants for age at first sex and birth linked to externalising behaviour. Nat Hum Behav.

