# Supplementary material for: Estimation of Machine Learning–Based Models to Predict Dementia Risk in Patients With Atherosclerotic Cardiovascular Diseases: UK Biobank Study
Source: JMIR Aging. 2025 Feb 26;8:e64148. doi: 10.2196/64148 (PMC11904384; doi:10.2196/64148)
Supplement: Multimedia Appendix 1 [file aging_v8i1e64148_app1.docx]

**Methods**

**Machine learning model (LightGBM)**

The LightGBM (Light Gradient Boosting Machine) algorithm is widely recognized as one of the most prevalent machine learning methodologies within the data science community.^1^ It represents an ensemble learning technique that relies on multiple base learners, typically decision tree models. LightGBM follows a boosting strategy, initially starting with a weak base learner (decision tree) and subsequently training each new tree to rectify errors made by prior trees. This sequential training process results in predictions that are aggregated to yield a robust final predictive model. During the validation phase, the algorithm calculates the probability output of classifying a participant into either category by averaging the outcomes derived from applying each individual decision tree to that participant. This approach leverages the strengths of ensemble learning to enhance predictive accuracy and generalization.

**Predictors importance ranking**

The predictor ranking was determined using an embedded function within the LightGBM algorithm. LightGBM is an ensemble tree-based model consisting of multiple decision tree models. A higher frequency of an attribute being used as a split node indicates its greater relative importance to the prediction model. These importance scores are explicitly calculated for each feature across the entire dataset, facilitating their ranking and comparison. Despite LightGBM being an ensemble method comprising numerous decision tree models, its performance heavily depends on adjustable hyperparameters. To mitigate potential biases arising from using a single set of hyperparameters, we trained 1,000 models across diverse parameter spaces. The top 5% of these models (50 out of 1,000) were selected based on their area under the ROC curves (AUCs). To ensure equal contributions from these 50 candidate models, their importance scores were normalized to a range of [0, 1]. Subsequently, the final importance ranking was computed by averaging the scores of these top-performing models and arranging them in descending order.

**Multicollinearity elimination**

The LightGBM algorithm demonstrates robust performance even when dealing with datasets afflicted by multicollinearity. This resilience stems from its approach of randomly selecting highly correlated features without bias towards any particular feature. However, this strategy can lead to challenges in interpreting the final selection of predictors, as many of them may belong to the same domain and offer similar or repetitive contributions to the model's predictions. To address this issue, we employed hierarchical clustering based on Spearman rank-order correlations.^2,3^ Through hierarchical clustering, predictors were grouped together if they exhibited high correlations. We then applied a threshold of 0.75 to the dendrogram, cutting it to retain only one predictor from each cluster below the threshold. This approach helped mitigate the interpretability issues caused by redundant predictors with high correlations.

**Hyperparameters tuning of the machine learning model**

The performance optimization of a LightGBM model is heavily dependent on hyperparameter tuning. We employed a 5-fold cross-validation strategy to identify the optimal parameter set. This involved iterating through the training process with 1,000 different hyperparameter configurations and selecting the best set based on the area under the ROC curve (AUC) metric. The hyperparameter search space used is detailed in Table S8. The final hyperparameters chosen to develop the machine learning (ML) model were as follows: "n_estimators": 2500, "num_leaves": 2010, "max_depth": 105, "subsample": 0.7, "learning_rate": 0.01, and "colsample_bytree": 0.7.

**Cross-validation evaluations**

We conducted a 5-fold cross-validation process by randomly dividing the dataset into five equal partitions or folds. In each iteration, four folds were designated for training the machine learning (ML) model, while the remaining fold served as the validation set. This process was repeated five times, with the folds shifting to ensure all data points were used for both training and validation. The training procedure comprised two stages: ML model establishment and output probability regression for calibration. The training data, consisting of four folds from the entire dataset, were split with a 3:1 ratio. Specifically, three folds were utilized for model development, while the remaining fold was reserved for calibration purposes. The validation sets were solely employed for evaluating the performance of the model. The reported results were calculated by averaging the statistics across the folds and presenting corresponding standard deviations for accuracy.

**Calibrations and predicted probabilities**

Calibration assesses the concordance between predicted probabilities and actual event proportions. This process involves sorting predicted probabilities and segmenting them into decile (10% quantile) groups. Within each subgroup, the average predicted probabilities were computed, while the observed event proportions were determined by dividing the number of events by the total number of participants. This methodology ensures that predicted probabilities exhibit a monotonically increasing trend, mirroring the anticipated distribution of observed proportions. The calibration goodness-of-fit was evaluated using the Kolmogorov-Smirnov test on the decile subgroups. A p-value greater than 0.05 signified an adequate goodness of fit. Our machine learning (ML) model was developed using data from the entire incident dementia population group of ASCVD patients and subsequently validated on distinct subsets (10-year /5-year incident dementia, 10-year/5-year incident AD and 10-year/5-year incident VD) (eFigure10-12).

**References**

1. Ke G, Meng Q, Finley T, et al. Lightgbm: A highly efficient gradient boosting decision tree. 2017 Presented at: 31st Conference on Neural Information Processing Systems. 2017:3149-57.

2. Han H, Oh J. Application of various machine learning techniques to predict obstructive sleep apnea syndrome severity. Sci Rep. 2023;13(1):6379.

3. Wang M, Yang B, Liu Y, Yang Y, Ji H, Yang C. Emerging infectious disease surveillance using a hierarchical diagnosis model and the Knox algorithm. Sci Rep. 2023;13(1):19836.

**Table S1. Codes used for three main types of dementia in the UK Biobank**

| **Diagnosis** | **Code Type** | **Codes** |
| --- | --- | --- |
| **ACD** | **ICD-9** | 290.2, 290.3, 290.4, 291.2, 294.1, 331.0, 331.1, 331.2, 331.5 |
|  | **ICD-10** | A81.0, F00, F00.0, F00.1, F00.2, F00.9, F01, F01.0, F01.1, F01.2, F01.3, F01.8, F01.9, F02, F02.0, F02.1, F02.2, F02.3, F02.4, F02.8, F03, F05.1, F10.6, G30, G30.0, G30.1, G30.8, G30.9, G31.0, G31.1, G31.8, I67.3 |
|  | **Read V2** | 1461, A411., A4110, E00.., E000., E001., E0010, E0011, E0012, E0013, E001z, E002., E0020, E0021, E002z, E003., E004., E0040, E0041, E0042, E0043, E004z, E012., E02y1, E041., Eu00., Eu000, Eu001, Eu002, Eu00z, Eu01., Eu010, Eu011, Eu012, Eu013, Eu01y, Eu01z, Eu02., Eu020, Eu021, Eu022, Eu023, Eu024, Eu025, Eu02y, Eu02z, Eu041, Eu106, Eu107, F110., F1100, F1101, F111., F112., F116., F118., F11x2, F11x7, F11x9, F11y2, F21y2, Fyu30, 38C13, 3AE3., 3AE4., 3AE5., 3AE6., 66h.., 6AB.., 8BM02, 8BM50, 8BM60, 8BPa., 8CMe0, 8CMG2, 8CMZ., 8CMZ0, 8CMZ1, 8CMZ2, 8CMZ3, 8CSA., 8Hla., 8IAe0, 8IAe2, 9hD.., 9hD0., 9hD1., 9Ou.., 9Ou1., 9Ou2., 9Ou3., 9Ou4., 9Ou5. |
|  | **Read** **CTV3** | .1461, 1461, .E11., .E111, .E112, .E113, .E114, .E115, .E116, .E11Z, .F21Z, .F371, .G78., A411., A4110, E00.., E000., E001., E0010, E0011, E0012, E0013, E001z, E002., E0020, E0021, E002z, E003., E004., E0040, E0041, E0042, E0043, E004z, E012., E02y1, E041., Eu00., Eu000, Eu001, Eu002, Eu00z, Eu01., Eu010, Eu011, Eu012, Eu013, Eu01y, Eu01z, Eu02., Eu020, Eu021, Eu022, Eu023, Eu024, Eu025, Eu02y, Eu02z, Eu041, F110., F1100, F1101, F111., F112., F116., F118., F11x2, F11x7, F11y2, F21y2, Fyu30, Ub1T6, X002m, X002w, X002x, X002y, X002z, X0030, X0031, X0032, X0033, X0034, X0035, X0036, X0037, X0039, X003A, X003B, X003C, X003D, X003E, X003F, X003G, X003H, X003I, X003J, X003l, X003m, X003P, X003R, X003T, X003V, X003W, X003X, X00R2, X00Rk, Xa0lH, Xa0sC, Xa0sE, Xa1GB, Xa25J, Xa3ez, XaA1S, XabVp, XaE74, XaIKB, XaIKC, XaKyY, XaOfZ, XE17j, XE1aG, XE1Xs, XE1Xu, XE1Z6, .3AE3, .3AE4, .3AE5, .3AE6, .66h., .6AB., .9hD1, .9Ou., .9Ou1, .9Ou2, .9Ou3, .9Ou4, .9Ou5, 3AE3., 3AE4., 3AE5., 3AE6., 66h.., 6AB.., 8BM02, 8BM50, 8BPa., 8CMe0, 8CMG2, 8CMZ., 8CMZ0, 8CMZ1, 8CMZ2, 8CMZ3, 8CSA., 8IAe0, 8IAe2, 9hD1., 9Ou., 9Ou1., 9Ou2., 9Ou3., 9Ou4., 9Ou5., Xa0fZ, XaaBZ, XaaeA, XaaiW, Xabd2, Xabd3, XabEk, XabEl, XabtQ, XacIx, XacIy, XacIz, XacJ0, XacLx, Xacly, Xaclz, XacM2, Xaefu, XaJBQ, XaJBU, XaJBV, XaJBW, XaJBX, XaJPy, XaLFf, XaLFo, XaLFp, XaMFy, XaMG0, XaMGF, XaMGG, XaMGI, XaMGJ, XaMGK, XaMJC, XaYFR, XaYPX, XaZqJ, XaZWz |
| **AD** | **ICD-9** | 331.0 |
|  | **ICD-10** | F00, F00.0, F00.1, F00.2, F00.9, G30, G30.0, G30.1, G30.8, G30.9 |
|  | **Read V2** | Eu00., Eu000, Eu001, Eu002, Eu00z, F110., F1100, F1101, Fyu30 |
|  | **Read CTV3** | .F21Z, Eu00., Eu000, Eu001, Eu002, Eu00z, F110., F1100, F1101, Fyu30, X002x, X002y, X002z, X0030, X0031, X0032, X0033, X003G, XaIKB, XaIKC, XE17j |
| **VD** | **ICD-9** | 290.4 |
|  | **ICD-10** | F01.0, F01.1, F01.2, F01.3, F01.8, F01.9 |
|  | **Read V2** | Eu01., E004, Eu011, Eu012, Eu012, Eu01y, Eu01z, E0040 |
|  | **Read CTV3** | Eu01., E004, Eu011, Eu012, Eu012, Eu01y, Eu01z, E0040 |

For detailed information, please refer to the website of UK Biobank (<https://www.ukbiobank.ac.uk>).

Abbreviations: ACD = All-Cause Dementia, AD = Alzheimer’s Disease, VD = Vascular Dementia, ICD = International Classification of Diseases, Read V2 = Read codes version 2, Read CTV3 = Read codes version 3.

**Table S2. List of variables used in the UKB in Machine Learning Models**

| **Main category** | **Field ID** |
| --- | --- |
| Population characteristic  (n = 2) | 31-0.0, 21022-0.0 |
| Cognitive function  (n = 22) | 398-0.1, 398-0.2, 399-0.1, 399-0.2, 400-0.1, 400-0.2, 403-0.0, 403-0.1, 403-0.2, 403-0.3, 403-0.4, 403-0.5, 403-0.6, 403-0.7, 403-0.8, 403-0.9, 403-0.10, 403-0.11, 404-0.7, 404-0.10, 404-0.11, 20023-0.0 |
| Physical measures  (n = 66) | 23098-0.0, 23099-0.0, 23100-0.0, 23101-0.0,23102-0.0,23104-0.0, 23105-0.0, 23106-0.0, 23107-0.0, 23108-0.0, 23109-0.0, 23110-0.0,23111-0.0, 23112-0.0, 23113-0.0, 23114-0.0, 23115-0.0, 23116-0.0, 23117-0.0, 23118-0.0, 23119-0.0, 23120-0.0, 23121-0.0, 23122-0.0, 23123-0.0, 23124-0.0, 23125-0.0, 23126-0.0, 23127-0.0, 23128-0.0, 23129-0.0, 23130-0.0, 48-0.0, 49-0.0, 50-0.0, 51-0.0, 3077-0.0, 20015-0.0, 21001-0.0, 21002-0.0, 102-0.0, 102-0.1, 4079-0.0, 4079-0.1, 4080-0.0, 4080-0.1, 3082-0.0, 46-0.0, 47-0.0, 3059-0.0, 3059-0.1, 3059-0.2, 3062-0.0, 3062-0.1, 3062-0.2, 3063-0.0, 3063-0.1, 3063-0.2, 3064-0.0, 3064-0.1, 3064-0.2, 20150-0.0, 20151-0.0, 20256-0.0, 20257-0.0, 20258-0.0 |
| Blood assays  (n=57) | 30610-0.0, 30620-0.0, 30640-0.0, 30650-0.0, 30660-0.0, 30670-0.0, 30680-0.0, 30690-0.0, 30700-0.0, 30710-0.0, 30720-0.0, 30730-0.0, 30740-0.0, 30750-0.0, 30760-0.0, 30770-0.0, 30780-0.0, 30790-0.0, 30810-0.0, 30830-0.0, 30840-0.0, 30850-0.0, 30860-0.0, 30870-0.0, 30880-0.0, 30890-0.0, 30000-0.0, 30010-0.0, 30020-0.0, 30030-0.0, 30040-0.0, 30050-0.0, 30060-0.0, 30070-0.0, 30080-0.0, 30090-0.0, 30100-0.0, 30110-0.0, 30120-0.0, 30130-0.0, 30140-0.0, 30150-0.0, 30160-0.0, 30170-0.0, 30180-0.0, 30190-0.0, 30200-0.0, 30210-0.0, 30220-0.0, 30230-0.0, 30240-0.0, 30250-0.0, 30260-0.0, 30270-0.0, 30280-0.0, 30290-0.0, 30300-0.0 |
| Urine assays  (n = 2) | 30520-0.0, 30530-0.0 |
| Touchscreen questionnaires  (n=151) | 1797-0.0, 1807-0.0, 1835-0.0, 1873-0.0, 1883-0.0, 3526-0.0, 20107-0.0, 20107-0.1, 20110-0.0, 20110-0.1, 20111-0.0, 20111-0.1, 2316-0.0, 2335-0.0, 2207-0.0, 2217-0.0, 2227-0.0, 2188-0.0, 2296-0.0, 2306-0.0, 2247-0.0, 2257-0.0, 3393-0.0, 2443-0.0, 2453-0.0, 2463-0.0, 2473-0.0, 2492-0.0, 1920-0.0, 1930-0.0, 1940-0.0, 1950-0.0, 1960-0.0, 1970-0.0, 1980-0.0, 1990-0.0, 2000-0.0, 2010-0.0, 2020-0.0, 2030-0.0, 2040-0.0, 2050-0.0, 2060-0.0, 2070-0.0, 2080-0.0, 2090-0.0, 2100-0.0, 20127-0.0, 87-0.0, 134-0.0, 135-0.0, 20008-0.0, 20009-0.0, 137-0.0, 136-0.0, 3079-0.0, 20010-0.0, 20011-0.0, 6142-0.0, 1558-0.0, 1568-0.0, 1578-0.0, 1588-0.0, 1598-0.0, 1608-0.0, 1628-0.0, 20117-0.0, 1289-0.0, 1299-0.0, 1309-0.0, 1319-0.0, 1329-0.0, 1339-0.0, 1349-0.0, 1359-0.0, 1369-0.0, 1379-0.0, 1389-0.0, 1428-0.0, 1438-0.0, 1448-0.0, 1458-0.0, 1468-0.0, 1478-0.0, 1488-0.0, 1498-0.0, 1508-0.0, 1518-0.0, 1528-0.0, 1538-0.0, 1548-0.0, 1110-0.0, 1120-0.0, 1130-0.0, 1140-0.0, 1150-0.0, 2237-0.0, 864-0.0, 874-0.0, 884-0.0, 894-0.0, 904-0.0, 924-0.0, 943-0.0, 971-0.0, 981-0.0, 1070-0.0, 1080-0.0, 1090-0.0, 1100-0.0, 22033-0.0, 22034-0.0, 22035-0.0, 22036-0.0, 22037-0.0, 22038-0.0, 22040-0.0, 2139-0.0, 2149-0.0, 2159-0.0, 1160-0.0, 1170-0.0, 1180-0.0, 1190-0.0, 1200-0.0, 1210-0.0, 1220-0.0, 1239-0.0, 1249-0.0, 1259-0.0, 1269-0.0, 1279-0.0, 20116-0.0, 20160-0.0, 1050-0.0, 1060-0.0, |
|  | 1717-0.0, 1727-0.0, 1737-0.0, 1747-0.0, 1757-0.0, 2267-0.0, 1031-0.0, 2110-0.0, 1677-0.0, 1687-0.0, 1697-0.0, 1707-0.0, 1767-0.0, 1777-0.0, 1787-0.0 |
| Self-generated variables  (n=16) | educ_yrs, fam_dem, diag_depress, activ, LegFatPercentageAvg, LegFatMassAvg, HandGripStrengthAvg, ImpedanceOfLegAvg, ImpedanceOfArmAvg, ImpedanceOfLimbAvg, FCV_Avg, PEF_Avg, Time2CompeleteRoundTotal, NbCorrectMatchesTotal, NbTimeSnapButtonPressedAvg, APOE4 gene types. |

A total of 16 predictors were manually generated, encompassing various factors necessary for deploying existing prediction scales, such as educ_yrs, fam_dem, diag_depress, depress_sym, and activ. These predictors were derived from existing diagnoses or self-reported health information. Additionally, predictors containing redundant information or multiple arrays were summarized using means or summations. This included predictors like LegFatPercentageAvg(average of leg fat percentage of left and right legs), LegFatMassAvg(average of leg fat mass of left and right legs), ImpedanceOfLegAvg(average impedance of left and right legs), ImpedanceOfArmAvg(average impedance of left and right arms), ImpedanceOfLimbAvg(average impedance of four limbs), HandGripStrengthAvg(average of hand grip strength of left and right hands), FCV_Avg(average of forced vital capacity in multiple arrays), PEF_Avg(average peak expiratory flow of multiple arrays), Time2CompleteRoundTotal(total time used to complete pairs matching game in all rounds), NbCorrectMatchesTotal(total number of corrected matches in pairs matching game), and NbTimeSnapButtonPressedAvg(average number of times snap-button pressed)., Finally, we also collected patients’ apolipoprotein E (ApoE) ε4 gene types. For more information of the remaining predictive factors, please refer to the website of UK Biobank.(<https://www.ukbiobank.ac.uk>).

**Table S3. The percentages of missing values for the top 10 selected predictors**

| **Variables** | **The proportion of missing values** |
| --- | --- |
| Forced vital capacity | 16.91% |
| Summed MET minutes per week for all activity | 22.35% |
| Age | 0.00% |
| Pairs matching time | 6.48% |
| Mean sphered cell volume | 6.47% |
| Glucose | 14.69% |
| Mean time to correctly identify matches | 1.73% |
| FEV1 z score | 32.60% |
| Age first had sexual intercourse | 13.04% |
| C-reactive protein | 7.16% |

Abbreviations: FEV1= Forced expiratory volume in one second, FVC= Forced vital capacity, MET= metabolic equivalent.

**Table S4. Top 25 predictors for all incident dementia with LightGBM after sensitivity analysis by MICE**

| **Number** | **Variables** | **Importance rating** | **Ranking** |
| --- | --- | --- | --- |
| 1 | Result ranking arraybest | 0.112 | 1 |
| 2 | Above moderate vigorous walking recommendation | 0.082 | 2 |
| 3 | Age | 0.073 | 3 |
| 4 | FEV1 | 0.041 | 4 |
| 5 | Pairs matching time (array 1) | 0.038 | 5 |
| 6 | Pairs matching time (total) | 0.031 | 6 |
| 7 | C-reactive protein | 0.031 | 7 |
| 8 | Glucose | 0.03 | 8 |
| 9 | Alcohol intake frequency | 0.03 | 9 |
| 10 | Gamma glutamyltransferase | 0.028 | 10 |
| 11 | FEV1 z score | 0.028 | 11 |
| 12 | Vitamin D | 0.027 | 12 |
| 13 | Mean corpuscular volume | 0.027 | 13 |
| 14 | HDL cholesterol | 0.026 | 14 |
| 15 | Total protein | 0.024 | 15 |
| 16 | Phosphate | 0.024 | 16 |
| 17 | Total bilirubin | 0.024 | 17 |
| 18 | Urate | 0.024 | 18 |
| 19 | Interpolated Year when non cancer illness first diagnosed | 0.024 | 19 |
| 20 | Result ranking array0 | 0.023 | 20 |
| 21 | Pulse rate automated reading array1 | 0.023 | 21 |
| 22 | Duration to first press of snap button in each round | 0.022 | 22 |
| 23 | Lipoprotein A | 0.022 | 23 |
| 24 | Father age at death | 0.022 | 24 |
| 25 | Red blood cell erythrocyte distribution width | 0.021 | 25 |

Abbreviations: FEV1= Forced expiratory volume in one second, FVC= Forced vital capacity, MET= metabolic equivalent. MICE = Multiple Imputation by Chained Equations.

**Tabel S5. Association between selected predictors and different types of dementia by Cox proportional hazards model**

|  | **Variables** | **Coef** | **SE** | **HR** | **P-value** |
| --- | --- | --- | --- | --- | --- |
| **All incident dementia** | Forced vital capacity | -0.092 | 0.098 | 0.91(0.75-1.10) | 0.352 |
|  | Summed MET minutes per week for all activity | -0.000 | 0.000 | 1.00(1.00-1.00) | 0.329 |
|  | Age | 0.122 | 0.018 | 1.10(1.10-1.20) | <0.001 |
|  | Pairs matching time | 0.001 | 0.000 | 1.00(1.00-1.00) | <0.001 |
|  | Mean sphered cell volume | 0.049 | 0.013 | 1.00(1.00-1.10) | <0.001 |
|  | Glucose | 0.104 | 0.035 | 1.10(1.00-1.20) | 0.003 |
|  | Mean time to correctly identify matches | 0.002 | 0.000 | 1.00(1.00-1.00) | <0.001 |
|  | FEV1 z score | 0.096 | 0.085 | 1.10(0.93-1.30) | 0.256 |
|  | Age first had sexual intercourse | -0.072 | 0.023 | 0.93(0.89-0.97) | 0.002 |
|  | C-reactive protein | -0.030 | 0.023 | 0.97(0.93-1.00) | 0.179 |
|  | Forced vital capacity | -0.092 | 0.098 | 0.91(0.75-1.10) | 0.352 |
| **All incident AD** | Summed minutes activity | 0.000 | 0.001 | 1.00(1.00-1.00) | 0.956 |
|  | Peak expiratory flow (PEF) | -0.000 | 0.001 | 1.00(1.00-1.00) | 0.861 |
|  | Pairs matching time | 0.000 | 0.000 | 1.00(1.00-1.00) | 0.023 |
|  | Age | 0.115 | 0.036 | 1.10(1.00-1.20) | 0.001 |
|  | Mean time to correctly identify matches | 0.002 | 0.000 | 1.00(1.00-1.00) | <0.001 |
|  | Glucose | 0.017 | 0.071 | 0.99(0.85-1.10) | 0.807 |
|  | Creatinin in urine | 0.000 | 0.000 | 1.00(1.00-1.00) | 0.199 |
|  | Apolipoprotein B | -0.409 | 0.661 | 0.92(0.31-2.80) | 0.536 |
|  | Mother age at death | -0.005 | 0.011 | 1.00(0.98-1.00) | 0.640 |
|  | Neuroticism score | 0.108 | 0.044 | 1.10(1.00-1.20) | 0.015 |
| **All incident VD** | Peak expiratory flow (PEF) | -0.000 | 0.001 | 1.00(1.00-1.00) | 0.800 |
|  | Summed minutes activity | -0.004 | 0.002 | 1.00(0.99-1.00) | 0.021 |
|  | Age | 0.108 | 0.029 | 1.10(1.10-1.20) | <0.001 |
|  | Glucose | 0.001 | 0.000 | 1.00(1.00-1.00) | 0.094 |
|  | FVC z score | 0.151 | 0.041 | 1.20(1.10-1.30) | <0.001 |
|  | Pairs matching time | 0.198 | 0.134 | 1.20(0.94-1.60) | 0.141 |
|  | Urea | -0.023 | 0.031 | 0.98(0.92-1.00) | 0.457 |
|  | Systolic blood pressure automated reading | 0.018 | 0.021 | 1.00(0.98-1.10) | 0.400 |
|  | Total bilirubin | -0.001 | 0.000 | 1.00(1.00-1.00) | 0.030 |
|  | Alkaline phosphatase | 0.011 | 0.006 | 1.00(1.00-1.00) | 0.091 |

Abbreviations: FEV1= Forced expiratory volume in one second, FVC= Forced vital capacity, MET= metabolic equivalent.

**Tabel S6. Association between selected predictors and AD by Cox proportional hazards model with competing risk of death**

| **AD** | **Without competing risk of death** | | | | **With competing risk of death** | | | |
| --- | --- | --- | --- | --- | --- | --- | --- | --- |
| **variables** | **Coef** | **Coef_se** | **HR** | **p_value** | **Coef** | **Coef_se** | **HR** | **p_value** |
| Summed minutes activity | -0.000 | 0.001 | 1.00(1.00-1.00) | 0.760 | -0.000 | 0.001 | 1.00(1.00-1.00) | 0.740 |
| Peak expiratory flow (PEF) | 0.000 | 0.001 | 1.00(1.00-1.00) | 0.985 | 0.000 | 0.000 | 1.00(1.00-1.00) | 0.980 |
| Pairs matching time | 0.000 | 0.000 | 1.00(1.00-1.00) | 0.002 | 0.000 | 0.000 | 1.00(1.00-1.00) | <0.001 |
| Age | 0.099 | 0.030 | 1.10(1.00-1.20) | 0.001 | 0.099 | 0.030 | 1.10(1.04-1.16) | 0.001 |
| Mean time to correctly identify matches | 0.002 | 0.000 | 1.00(1.00-1.00) | 0.005 | 0.002 | 0.000 | 1.00(1.00-1.00) | 0.008 |
| Glucose | -0.013 | 0.076 | 0.99(0.85-1.10) | 0.866 | -0.013 | 0.064 | 0.99(0.86-1.11) | 0.840 |
| Creatinin in urine | 0.000 | 0.000 | 1.00(1.00-1.00) | 0.157 | 0.000 | 0.000 | 1.00(1.00-1.00) | 0.120 |
| Apolipoprotein B | -0.080 | 0.564 | 0.92(0.31-2.80) | 0.887 | -0.080 | 0.574 | 0.92(-0.20-2.05) | 0.890 |
| Mother age at death | -0.002 | 0.010 | 1.00(0.98-1.00) | 0.875 | -0.002 | 0.010 | 1.00(0.98-1.02) | 0.870 |
| Neuroticism score | 0.079 | 0.039 | 1.10(1.00-1.20) | 0.042 | 0.079 | 0.039 | 1.08(1.01-1.16) | 0.043 |

After competing risk analysis of death, the association between selected predictors and AD did not change with Cox proportional hazards model.

**Tabel S7. Association between selected predictors and VD by Cox proportional hazards model with competing risk of death**

| **VD** | **Without competing risk of death** | | | | **With competing risk of death** | | | |
| --- | --- | --- | --- | --- | --- | --- | --- | --- |
| **variables** | **Coef** | **Coef_se** | **HR** | **p_value** | **Coef** | **Coef_se** | **HR** | **p_value** |
| Summed minutes activity | -0.002 | 0.001 | 1.00(1.00-1.00) | 0.022 | -0.002 | 0.001 | 1.00(1.00-1.00) | 0.028 |
| Peak expiratory flow (PEF) | -0.003 | 0.001 | 1.00(0.99-1.00) | 0.025 | -0.003 | 0.001 | 1.00(1.00-1.00) | 0.029 |
| Pairs matching time | 0.108 | 0.025 | 1.10(1.10-1.20) | <0.001 | 0.108 | 0.027 | 1.11(1.06-1.17) | <0.001 |
| Age | 0.123 | 0.035 | 1.10(1.10-1.20) | <0.001 | 0.123 | 0.038 | 1.13(1.06-1.21) | 0.001 |
| Mean time to correctly identify matches | 0.018 | 0.111 | 1.00(0.82-1.30) | 0.873 | 0.018 | 0.123 | 1.02(0.78-1.26) | 0.890 |
| Glucose | 0.001 | 0.000 | 1.00(1.00-1.00) | <0.001 | 0.001 | 0.000 | 1.00(1.00-1.00) | <0.001 |
| Creatinin in urine | -0.080 | 0.071 | 0.92(0.80-1.10) | 0.263 | -0.080 | 0.092 | 0.92(0.74-1.10) | 0.390 |
| Apolipoprotein B | 0.008 | 0.006 | 1.00(1.00-1.00) | 0.154 | 0.008 | 0.007 | 1.01(1.00-1.02) | 0.230 |
| Mother age at death | -0.016 | 0.026 | 0.98(0.94-1.00) | 0.543 | -0.016 | 0.036 | 0.98(0.91-1.05) | 0.660 |
| Neuroticism score | 0.002 | 0.003 | 1.00(1.00-1.00) | 0.564 | 0.002 | 0.004 | 1.00(0.99-1.01) | 0.640 |

After competing risk analysis of death, the association between selected predictors and VD did not change with Cox proportional hazards model.

**Table S8. Hyperparameter space explored for LightGBM classifier**

| **Hyperparameters** | **Search space** | **Step** | **Final choice** |
| --- | --- | --- | --- |
| n_estimators | 100,200,300,400 | 100 | 200 |
| num_leaves | 20,40,60,80 | 20 | 20 |
| max_depth | 5,10,15,20 | 5 | 10 |
| subsample | 0.7,0.8,0.9 | 0.1 | 0.7 |
| colsample_bytree | 0.7,0.8,0.9 | 0.1 | 0.7 |
| learning_rate | 0.001,0.01,0.1 | 10 | 0.1 |

The table above illustrates the utilized parameters for the LGBM algorithm. Further information regarding the parameters can be accessed at https://lightgbm.readthedocs.io/en/v3.3.2/Parameters.htm.

**Figure S1. Performance for the prediction on different types of dementia by Cox proportional hazards model**


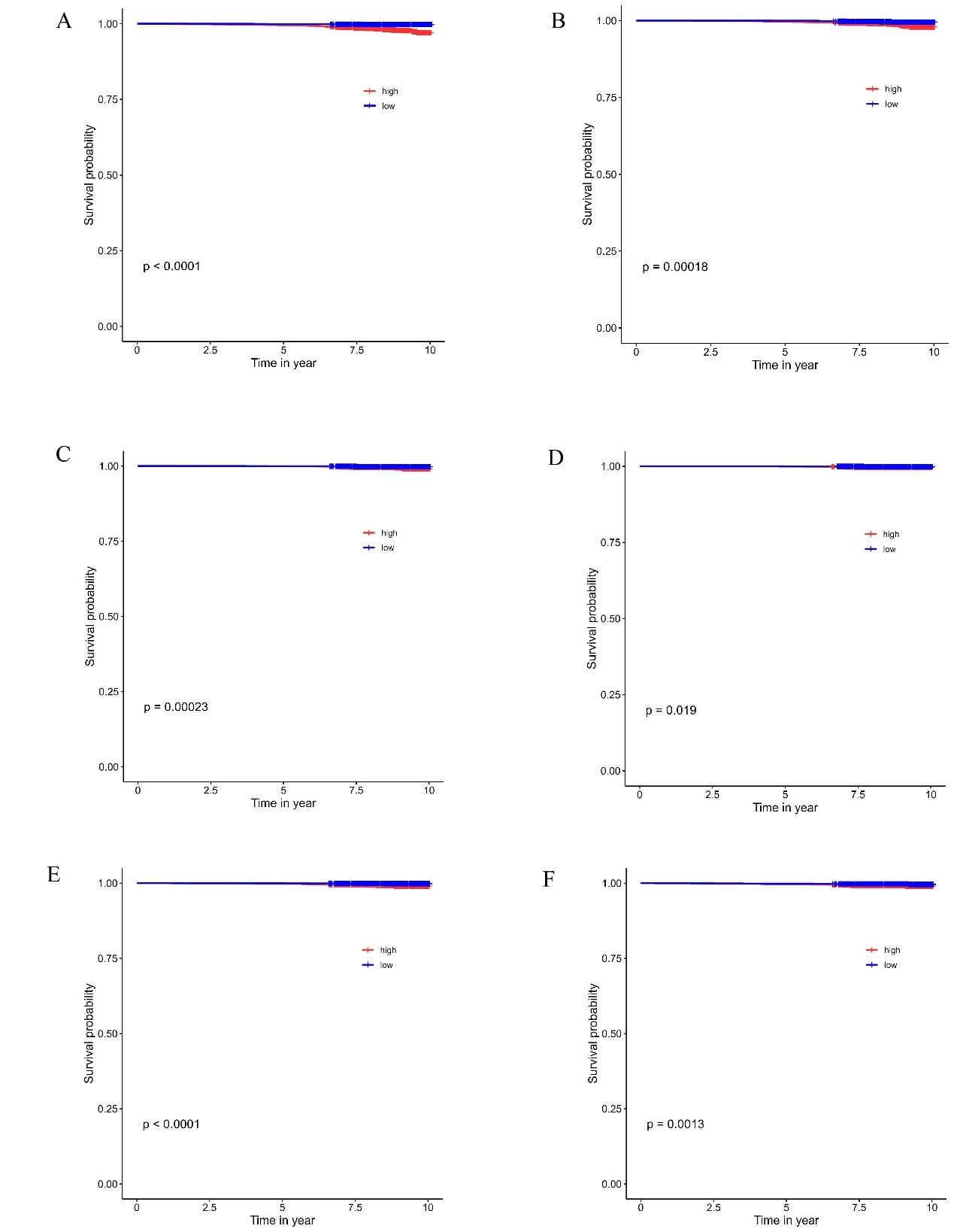


(A-B) Kaplan-Meier survival curve analysis of all incident dementia in the train data and test data; (C-D) Kaplan-Meier survival curve analysis of all incident AD in the train data and test data; (E-F) Kaplan-Meier survival curve analysis of all incident VD in the train data and test data. Abbreviations: AD = Alzheimer’s Disease, VD = Vascular Dementia.

**Figure S2. Performance for the prediction on AD and VD by Cox proportional hazards model with competing risk of death**


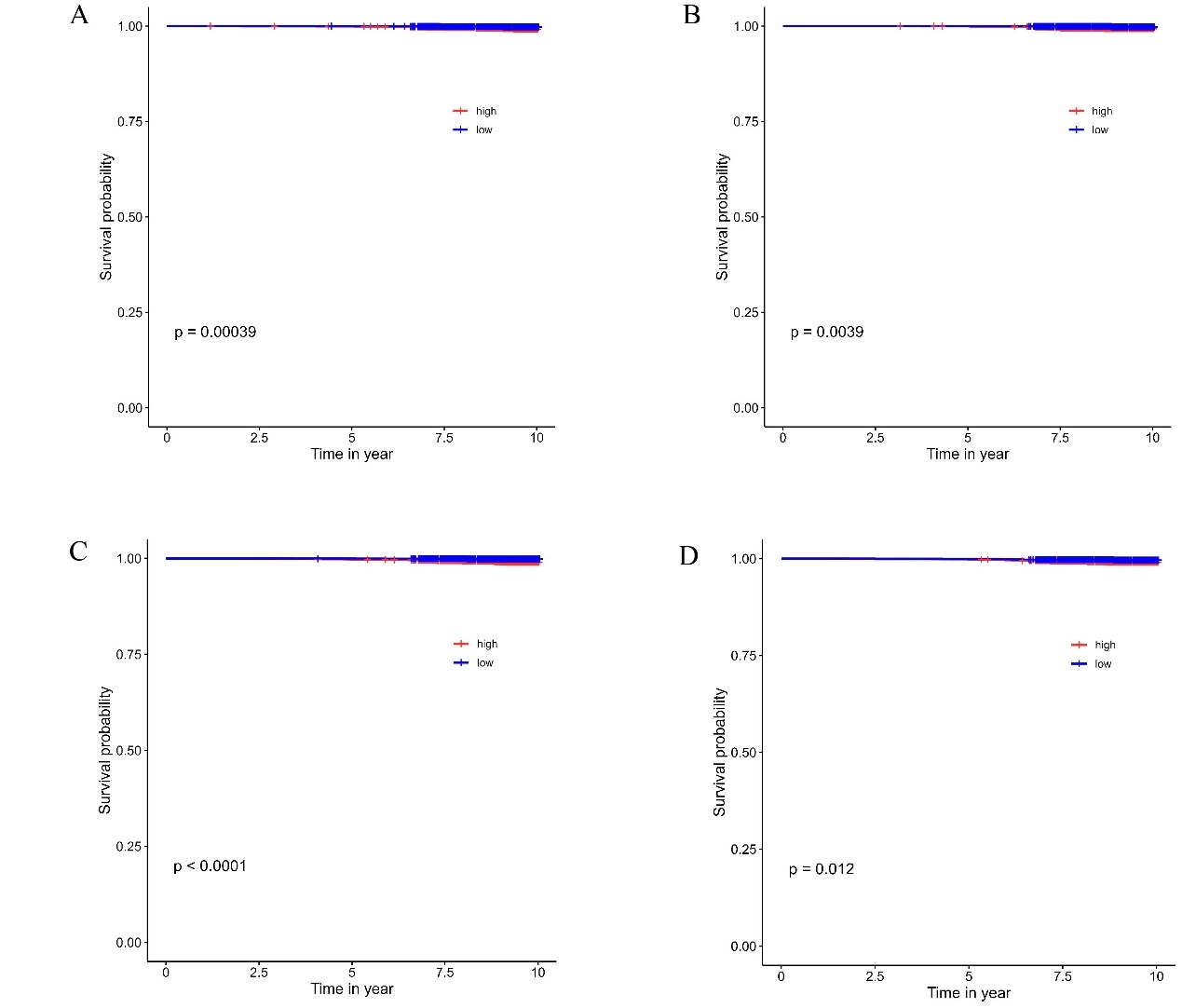


(A-B) Kaplan-Meier survival curve analysis of all incident AD in the train data and test data with competing risk analysis; (C-D) Kaplan-Meier survival curve analysis of all incident VD in the train data and test data with competing risk analysis. Abbreviations: AD = Alzheimer’s Disease, VD = Vascular Dementia.

**Figure S3. Predictive variables selection and interpretation on all incident dementia**


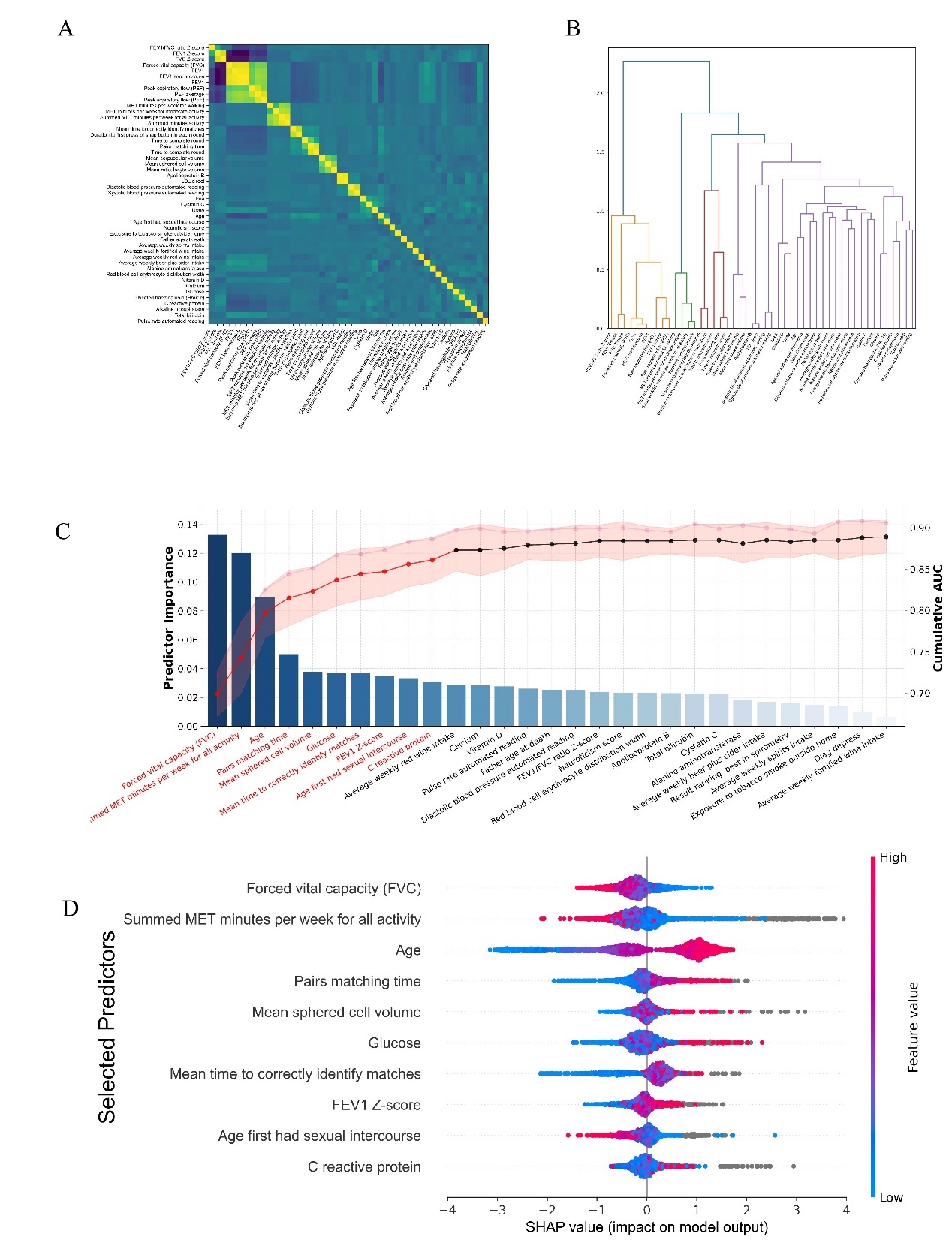


(A) Heatmap depicting Spearman rank-order correlations among the top-50 candidate predictors for all incident dementia population modeling; (B) Hierarchical clustering dendrogram constructed using calculated correlations; (C) Sequential forward selection from a preselected predictor pool; (D) SHAP-based Visualization of Salient Predictors.

Abbreviations: FEV1 = forced expiratory volume in one second; FVC = forced vital capacity; MET = metabolic equivalent; SHAP = SHapley Additive exPlanations.

**Figure S4. Predictive variables selection and interpretation on 10-year incident dementia**


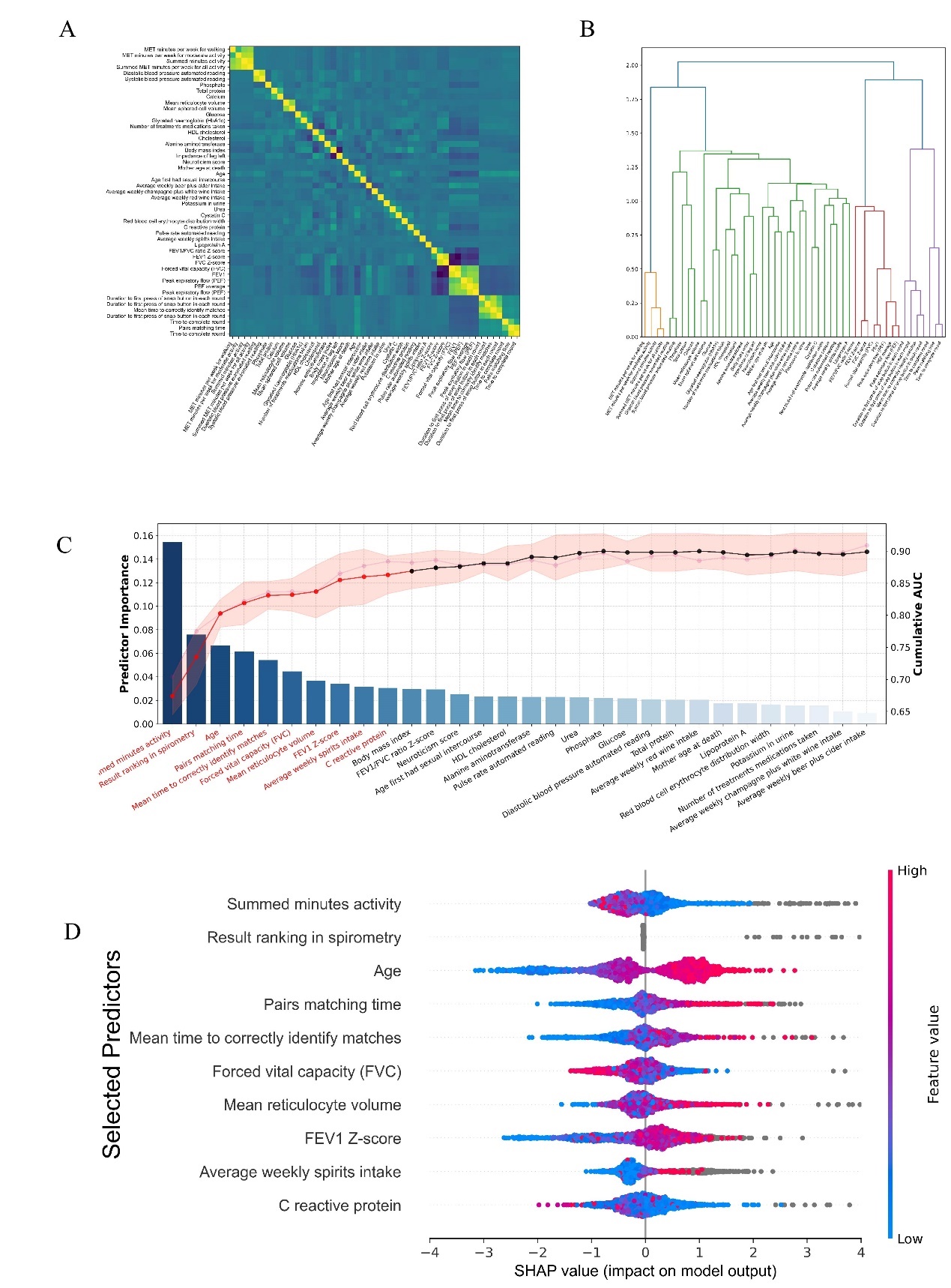


(A) Heatmap depicting Spearman rank-order correlations among the top-50 candidate predictors for 10-year incident dementia population modeling; (B) Hierarchical clustering dendrogram constructed using calculated correlations; (C) Sequential forward selection from a preselected predictor pool; (D) SHAP-based Visualization of Salient Predictors.

Abbreviations: FEV1 = forced expiratory volume in one second; FVC = forced vital capacity; MET = metabolic equivalent; SHAP = SHapley Additive exPlanations.

**Figure S5. Predictive variables selection and interpretation on 5-year incident dementia**


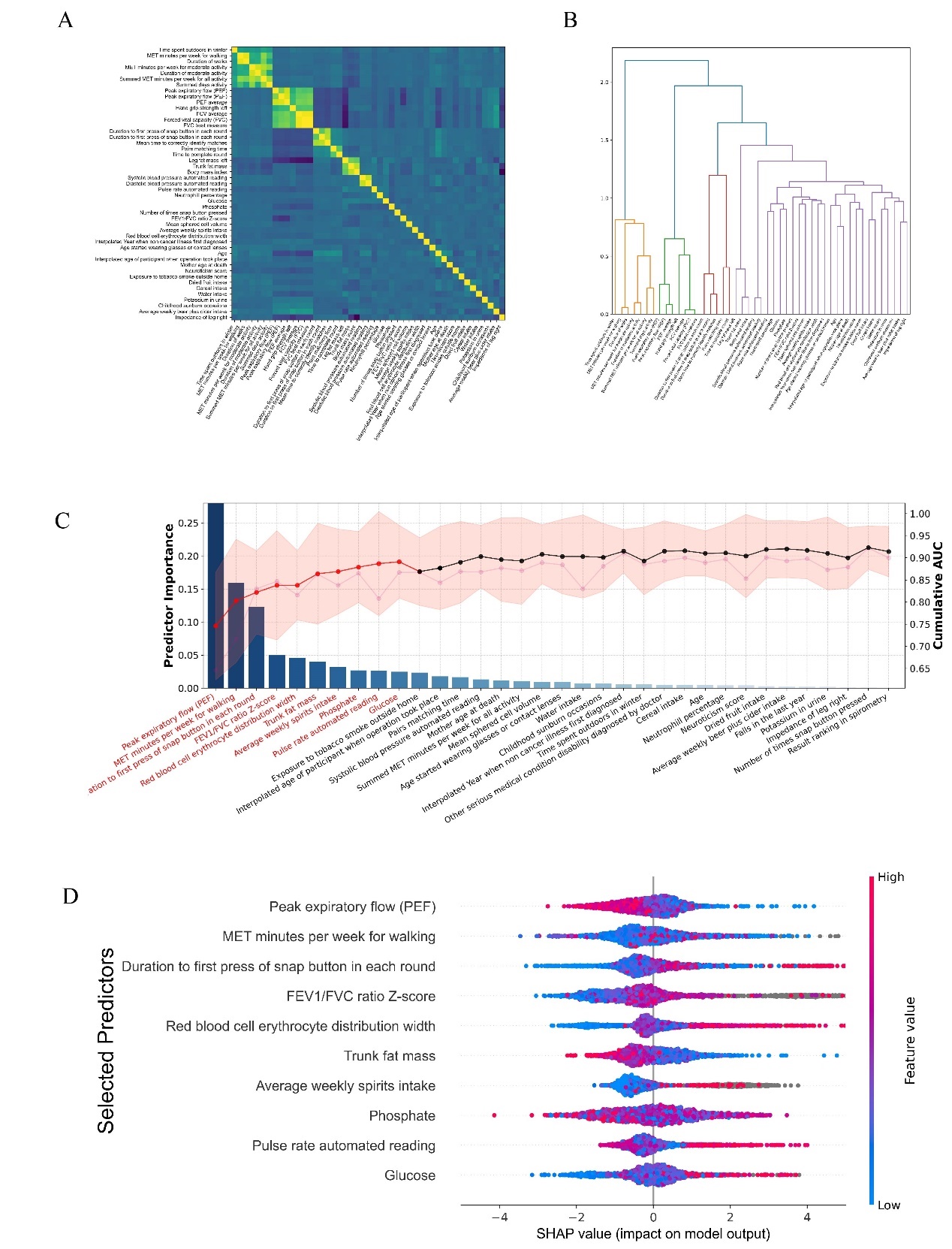


(A) Heatmap depicting Spearman rank-order correlations among the top-50 candidate predictors for 5-year incident dementia population modeling; (B) Hierarchical clustering dendrogram constructed using calculated correlations; (C) Sequential forward selection from a preselected predictor pool; (D) SHAP-based Visualization of Salient Predictors.

Abbreviations: FEV1 = forced expiratory volume in one second; FVC = forced vital capacity; MET = metabolic equivalent; SHAP = SHapley Additive exPlanations.

**Figure S6. Predictive variables selection and interpretation on all incident AD**


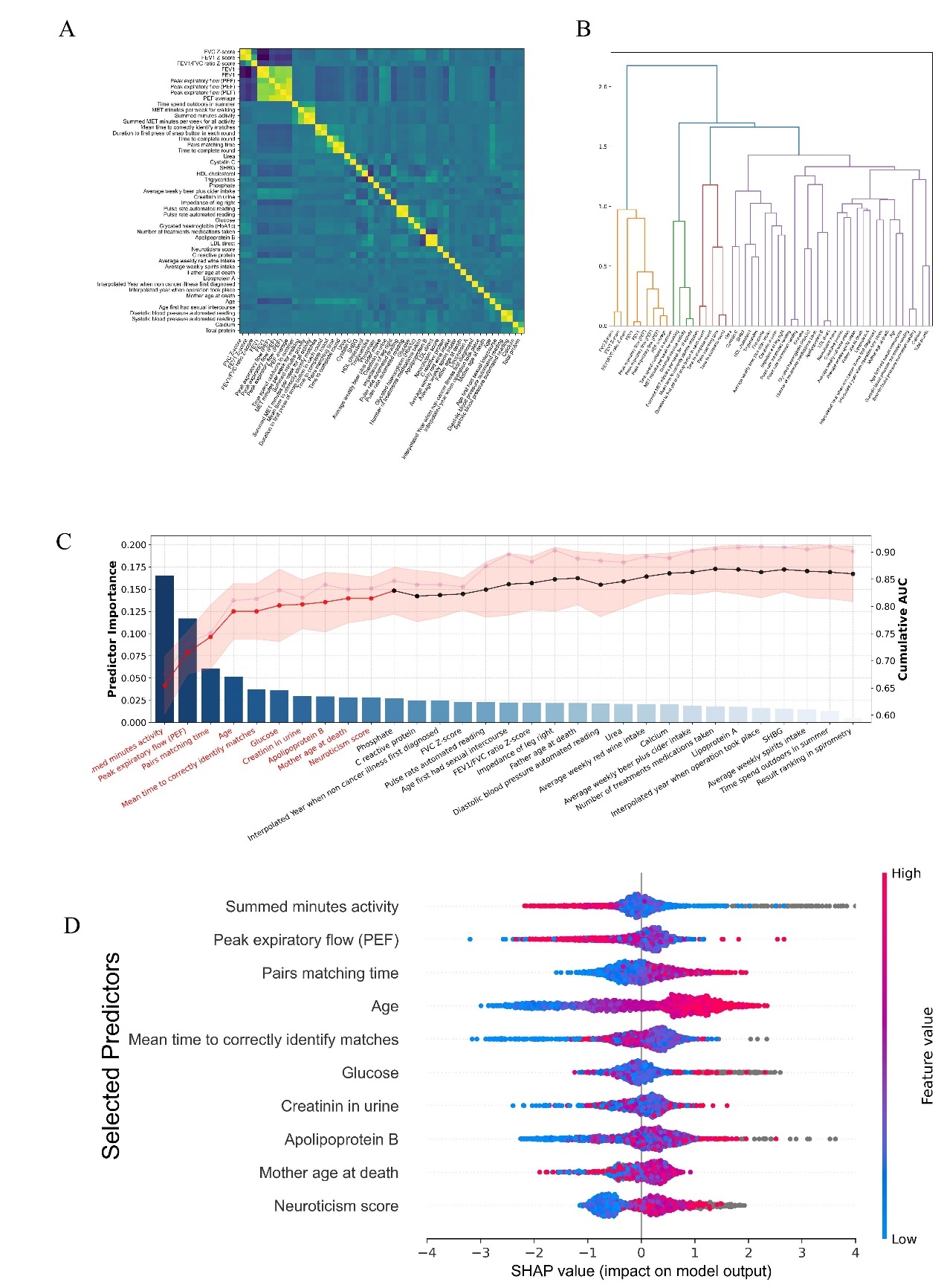


(A) Heatmap depicting Spearman rank-order correlations among the top-50 candidate predictors for all incident AD population modeling; (B) Hierarchical clustering dendrogram constructed using calculated correlations; (C) Sequential forward selection from a preselected predictor pool; (D) SHAP-based Visualization of Salient Predictors.

Abbreviations: SHAP = SHapley Additive exPlanations.

**Figure S7. Predictive variables selection and interpretation on 10-year incident AD**


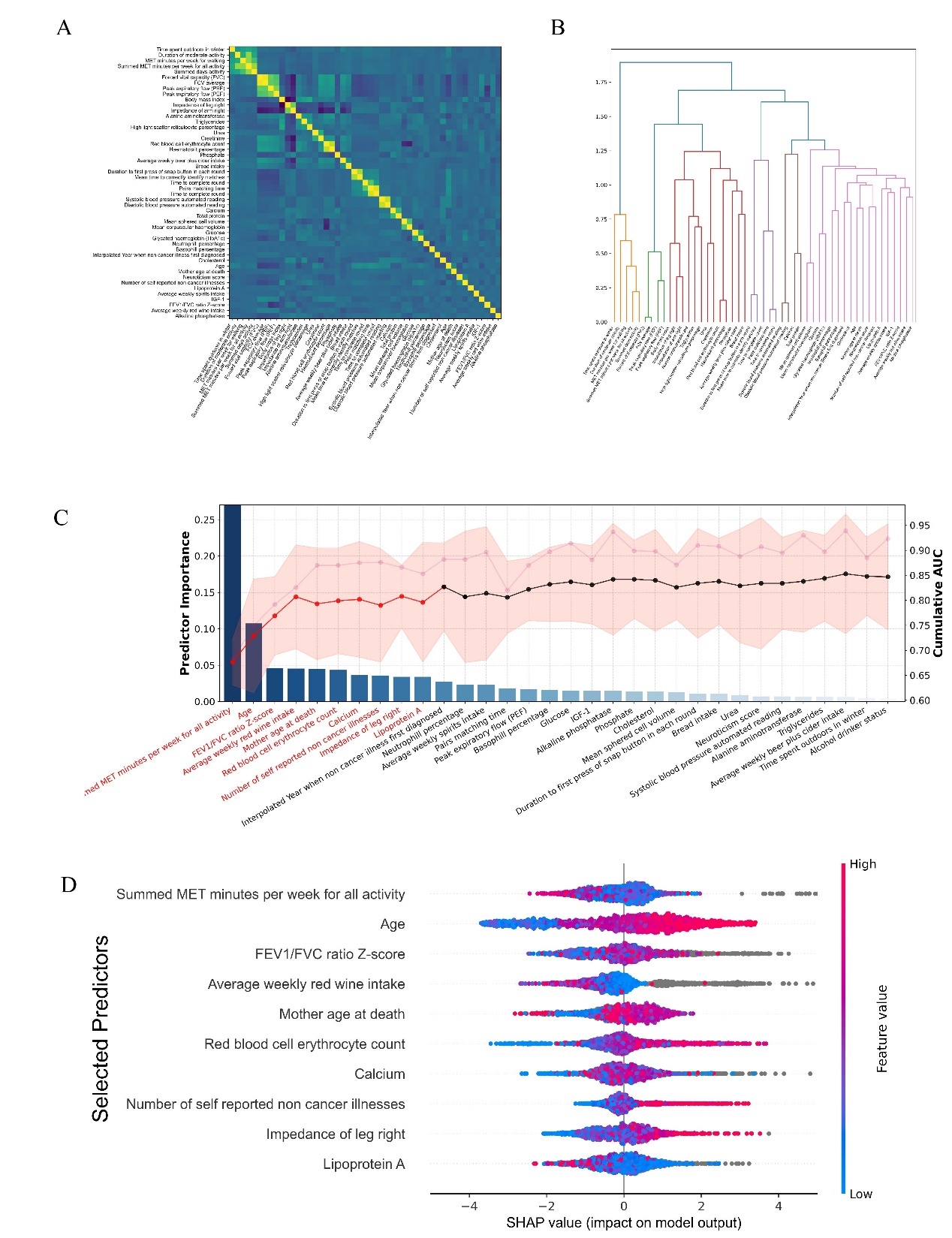


(A) Heatmap depicting Spearman rank-order correlations among the top-50 candidate predictors for 10-year incident AD population modeling; (B) Hierarchical clustering dendrogram constructed using calculated correlations; (C) Sequential forward selection from a preselected predictor pool; (D) SHAP-based Visualization of Salient Predictors.

Abbreviations: FEV1 = forced expiratory volume in one second; FVC = forced vital capacity; MET = metabolic equivalent; SHAP = SHapley Additive exPlanations.

**Figure S8. Predictive variables selection and interpretation on 5-year incident AD**


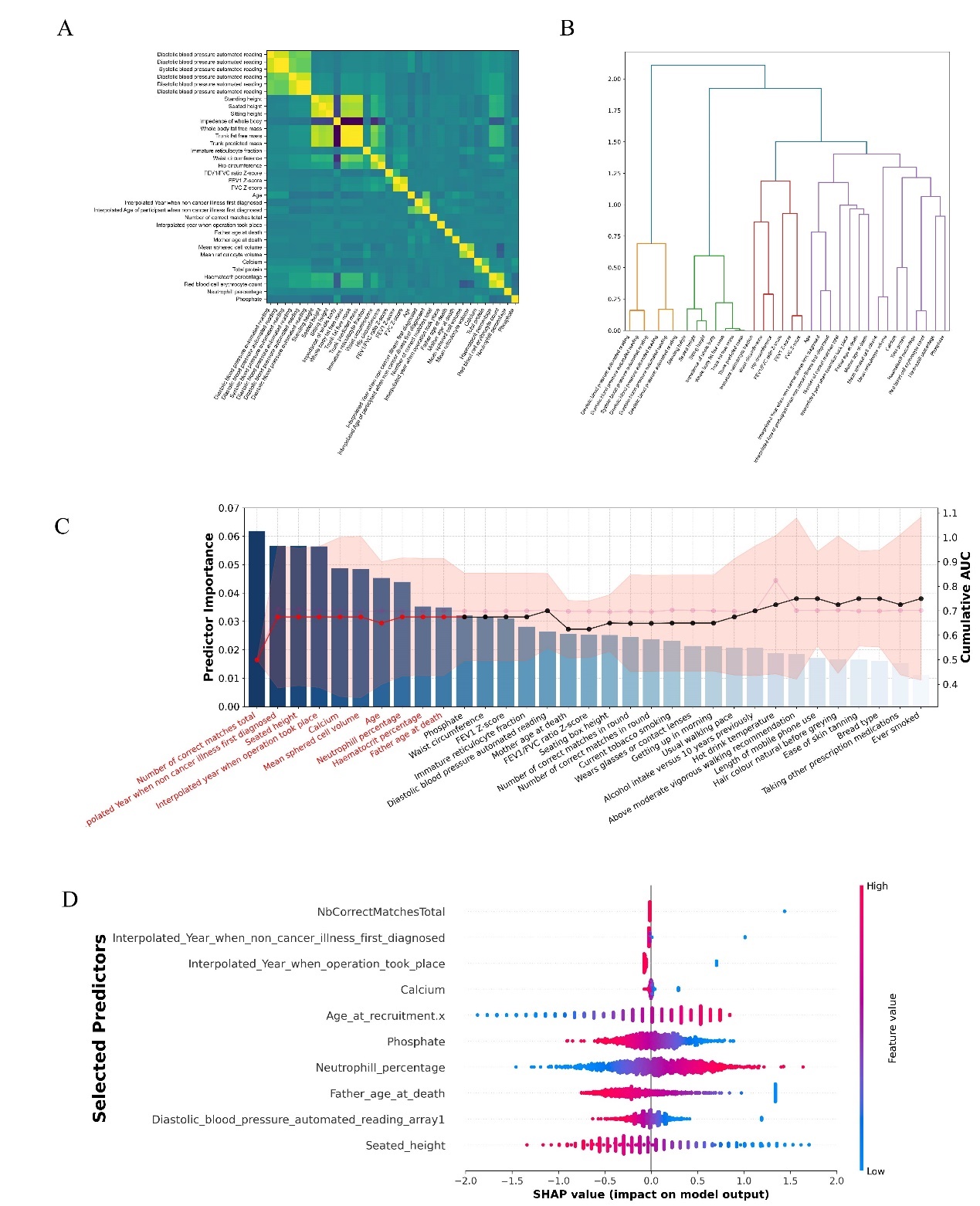


(A) Heatmap depicting Spearman rank-order correlations among the top-50 candidate predictors for 5-year incident AD population modeling; (B) Hierarchical clustering dendrogram constructed using calculated correlations; (C) Sequential forward selection from a preselected predictor pool; (D) SHAP-based Visualization of Salient Predictors.

Abbreviations: SHAP = SHapley Additive exPlanations.

**Figure S9. Predictive variables selection and interpretation on all incident VD**


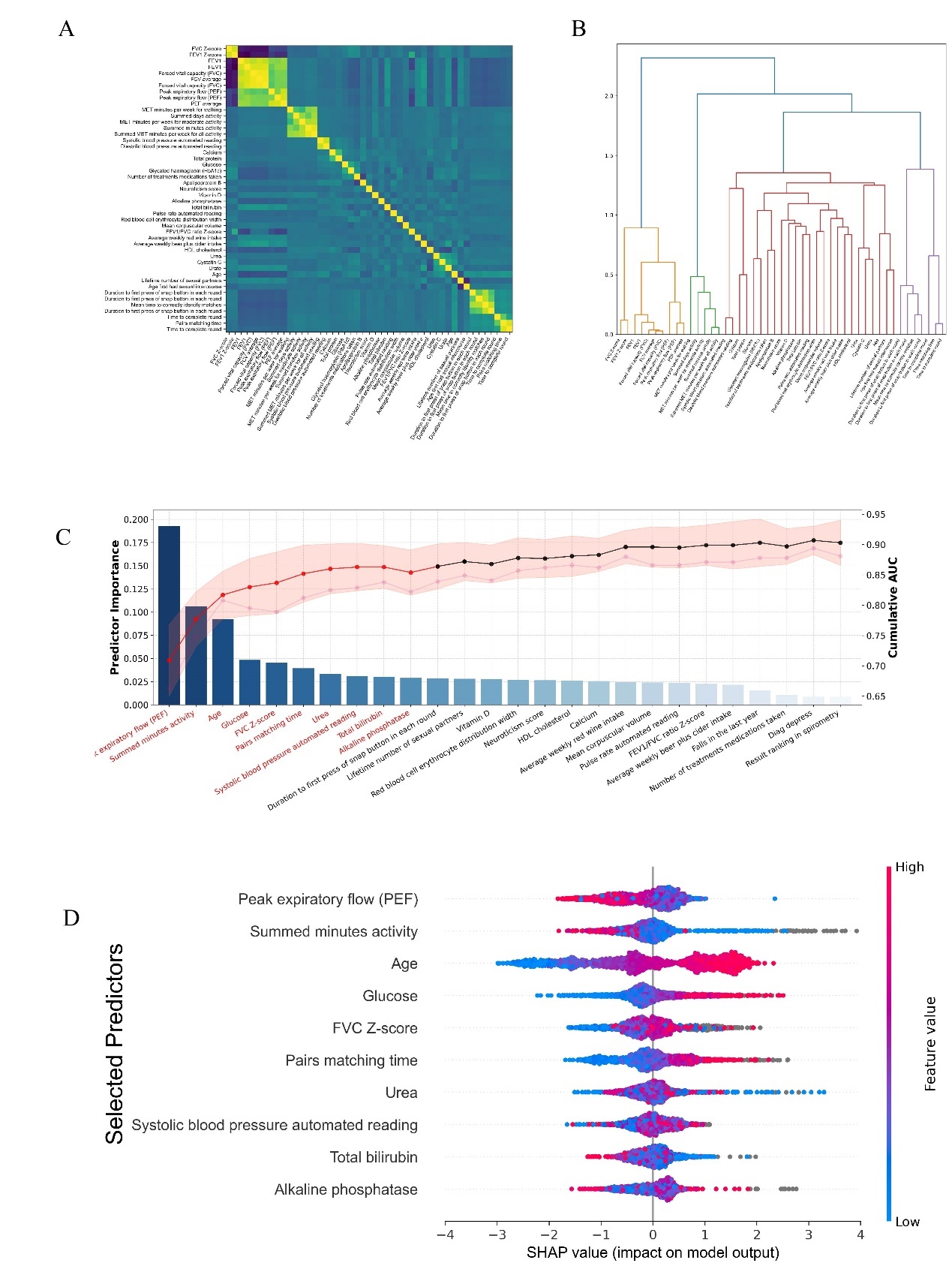


(A) Heatmap depicting Spearman rank-order correlations among the top-50 candidate predictors for all incident VD population modeling; (B) Hierarchical clustering dendrogram constructed using calculated correlations; (C) Sequential forward selection from a preselected predictor pool; (D) SHAP-based Visualization of Salient Predictors.

Abbreviations: FVC = forced vital capacity; SHAP = SHapley Additive exPlanations.

**Figure S10. Predictive variables selection and interpretation on 10-year incident VD**


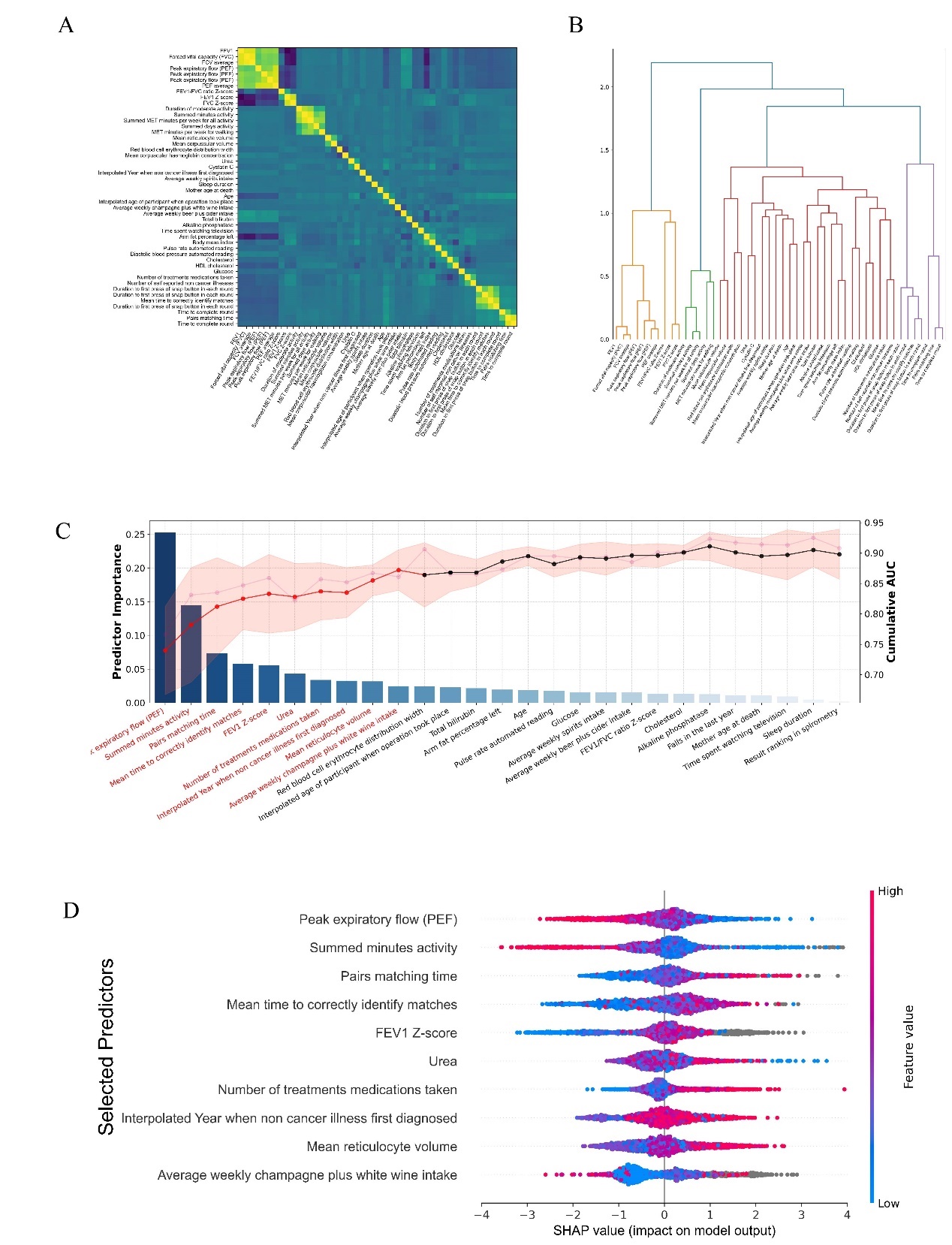


(A) Heatmap depicting Spearman rank-order correlations among the top-50 candidate predictors for 10-year incident VD population modeling; (B) Hierarchical clustering dendrogram constructed using calculated correlations; (C) Sequential forward selection from a preselected predictor pool; (D) SHAP-based Visualization of Salient Predictors.

Abbreviations: FEV1 = forced expiratory volume in one second; SHAP = SHapley Additive exPlanations.

**Figure S11. Predictive variables selection and interpretation on 5-year incident VD**


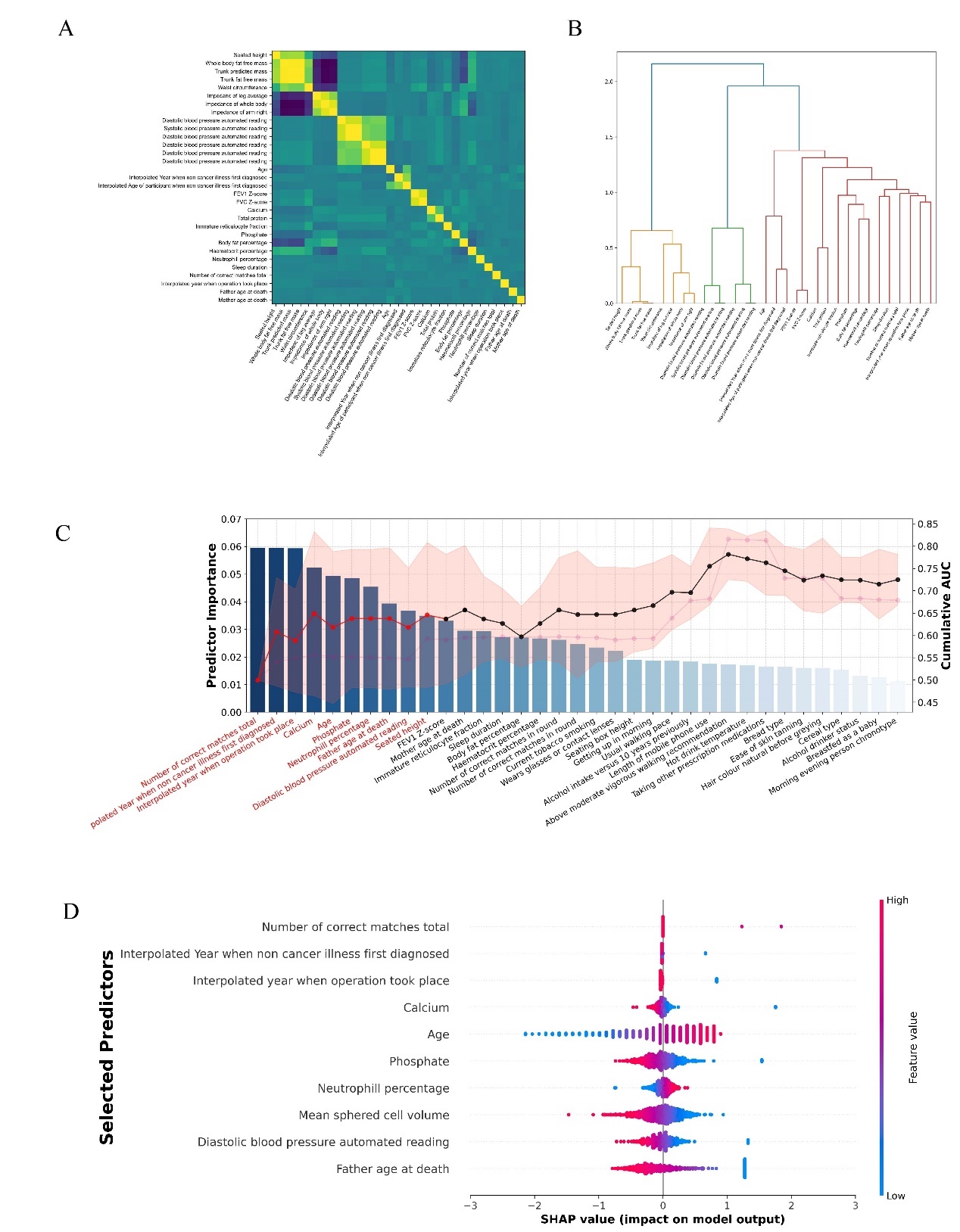


(A) Heatmap depicting Spearman rank-order correlations among the top-50 candidate predictors for 5-year incident VD population modeling; (B) Hierarchical clustering dendrogram constructed using calculated correlations; (C) Sequential forward selection from a preselected predictor pool; (D) SHAP-based Visualization of Salient Predictors.

Abbreviations: SHAP = SHapley Additive exPlanations.

**Figure S12. Calibration plots of incident dementia at different timelines**


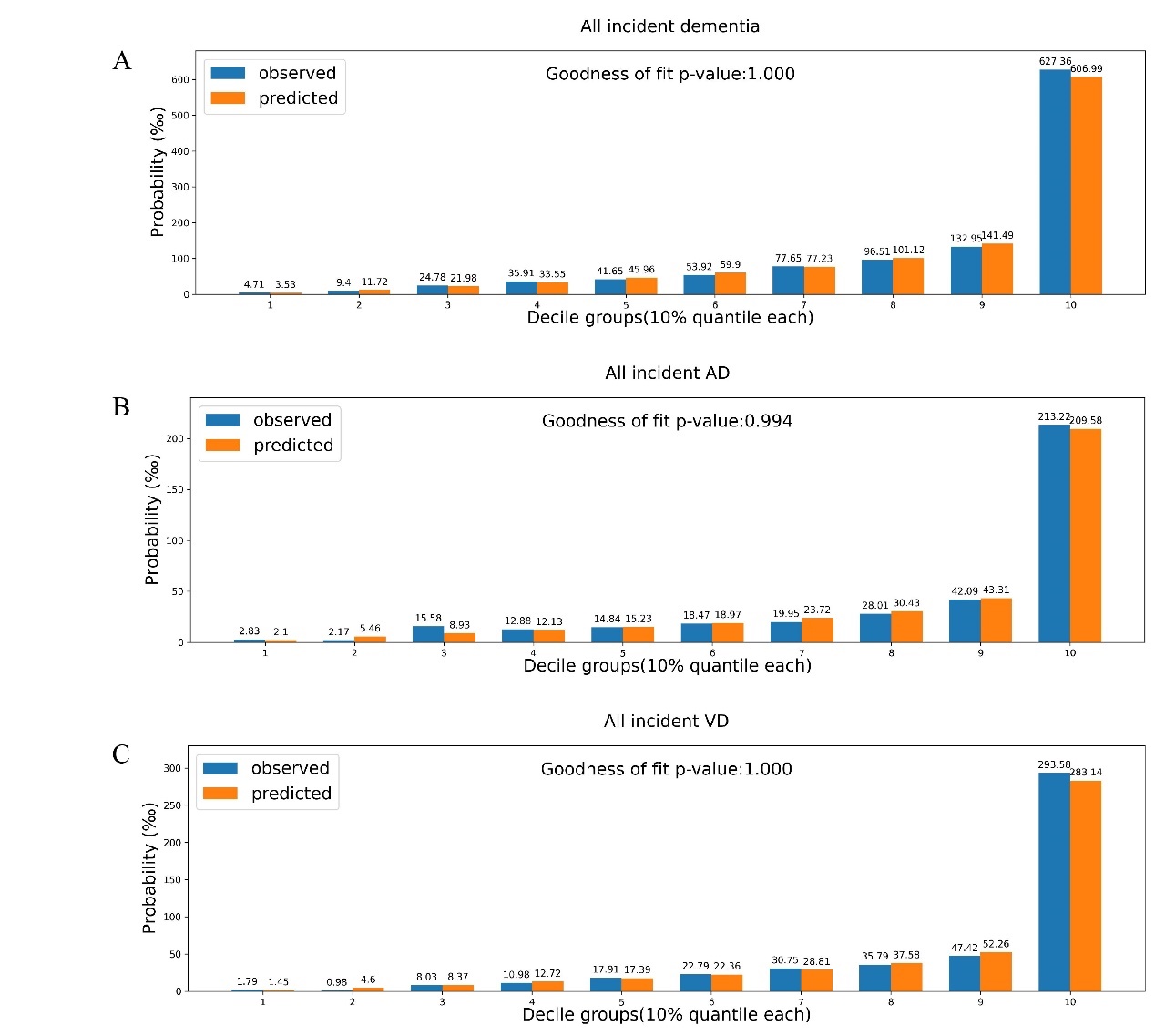


All calibrations were plotted using raw predicted probabilities adjusted to observed all incident dementia events. Our model exhibited satisfactory calibrations with a p-value > 0.05.

**Figure S13. Calibration plots of incident AD at different timelines**


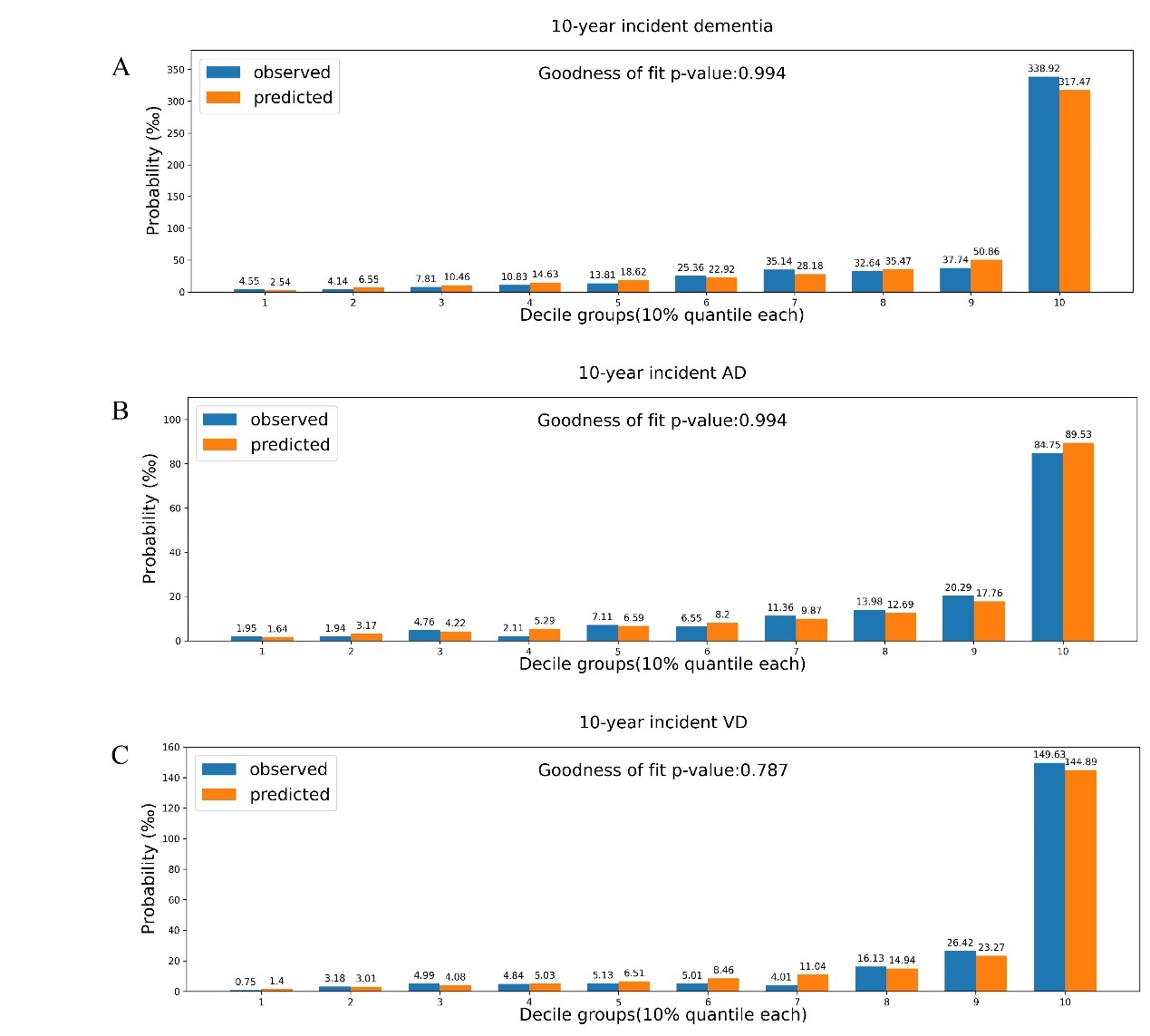


All calibrations were plotted using raw predicted probabilities adjusted to observed all incident AD events. Our model exhibited satisfactory calibrations with a p-value > 0.05.

Abbreviations: AD = Alzheimer’s Disease.

**Figure S14. Calibration plots of incident VD at different timelines**


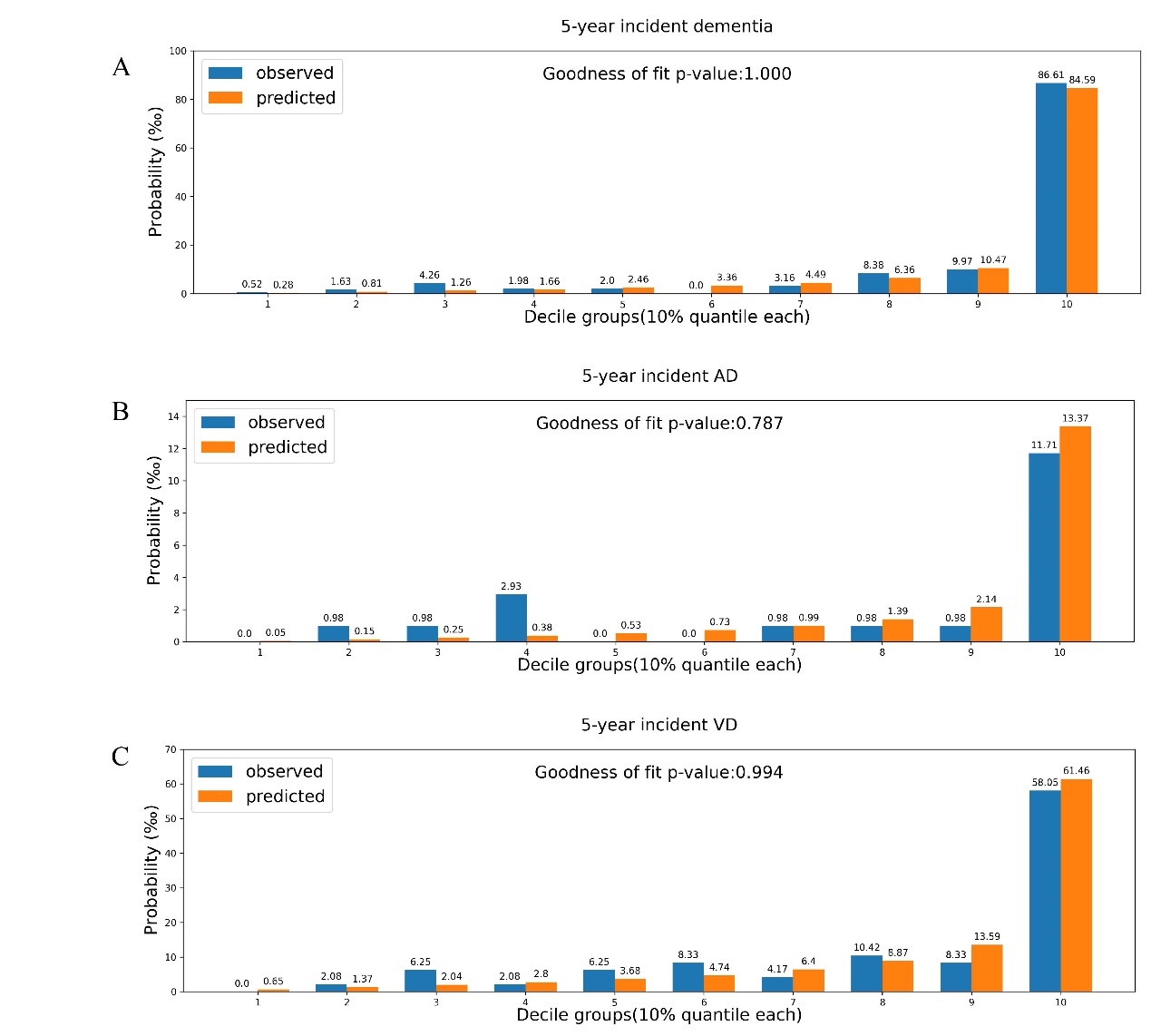


All calibrations were plotted using raw predicted probabilities adjusted to observed all incident VD events. Our model exhibited satisfactory calibrations with a p-value > 0.05.

Abbreviations: VD= Vascular Dementia.

**Figure S15. Correlation heatmap of included predictors**


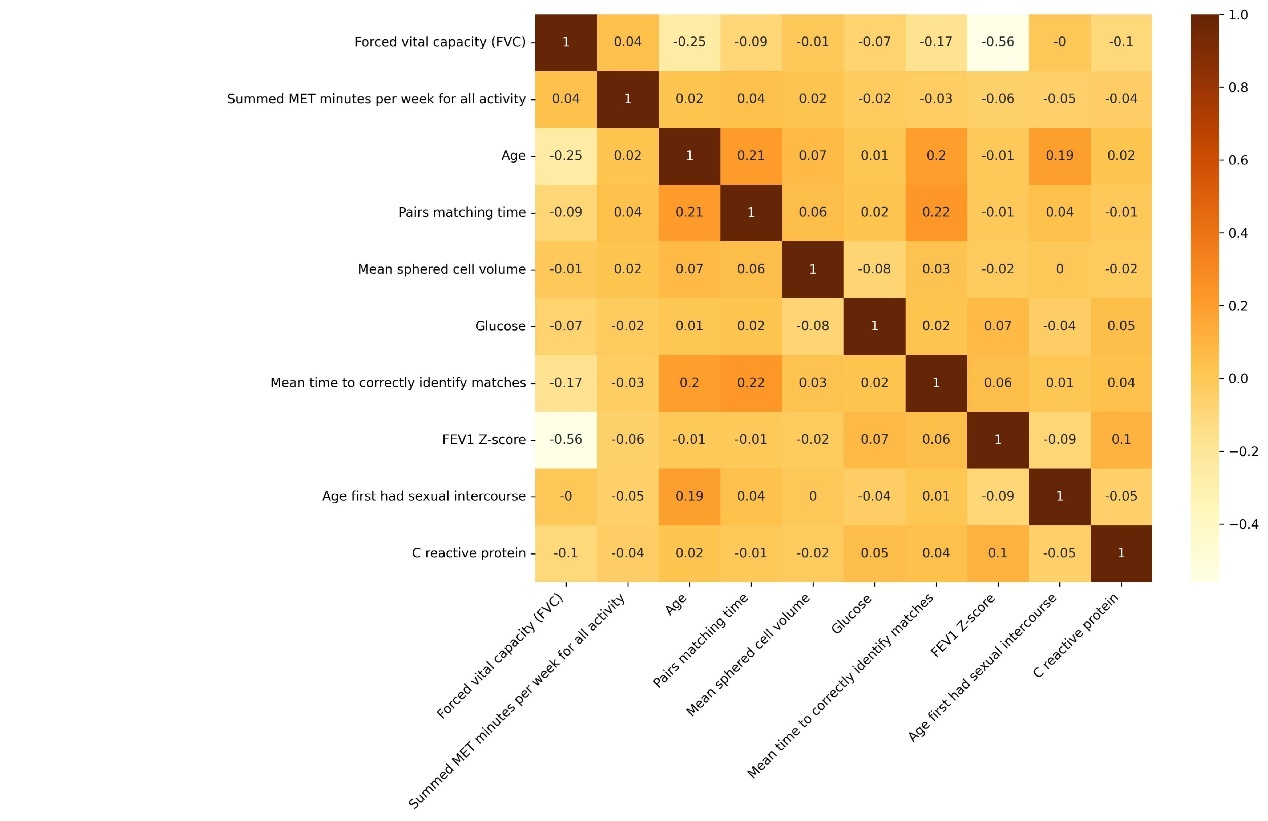


Abbreviations: FEV1 = forced expiratory volume in one second; FVC = forced vital capacity; MET = metabolic equivalent.
